# Supplementary figures and images for: Apoptotic signaling clears engineered Salmonella in an organ-specific manner
Source: eLife. 2023 Dec 6;12:RP89210. doi: 10.7554/eLife.89210 (PMC10699806; doi:10.7554/eLife.89210)

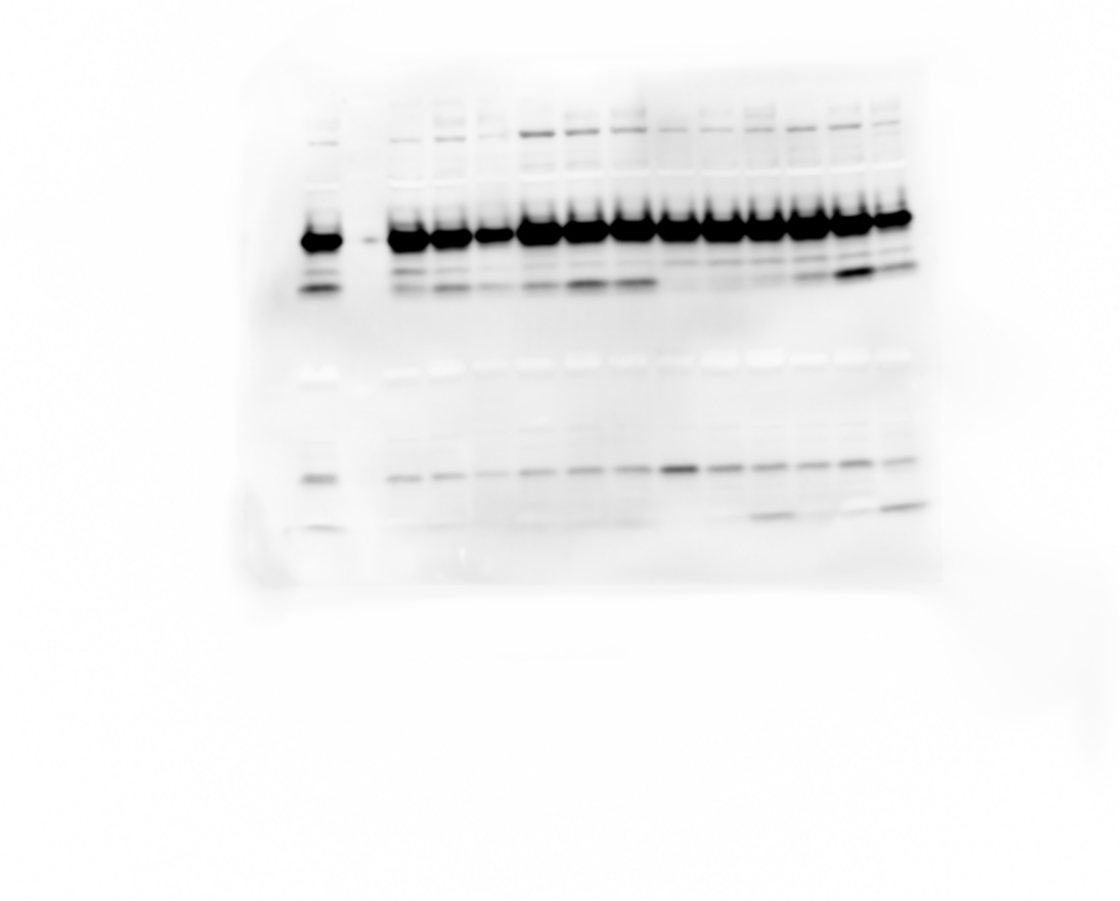

Supplement: Figure 1—source data 1. [file elife-89210-fig1-data1.zip › Figure 1- Source Data 1/individual images/BID_5_min_exposure_pico_gamma_.jpg]

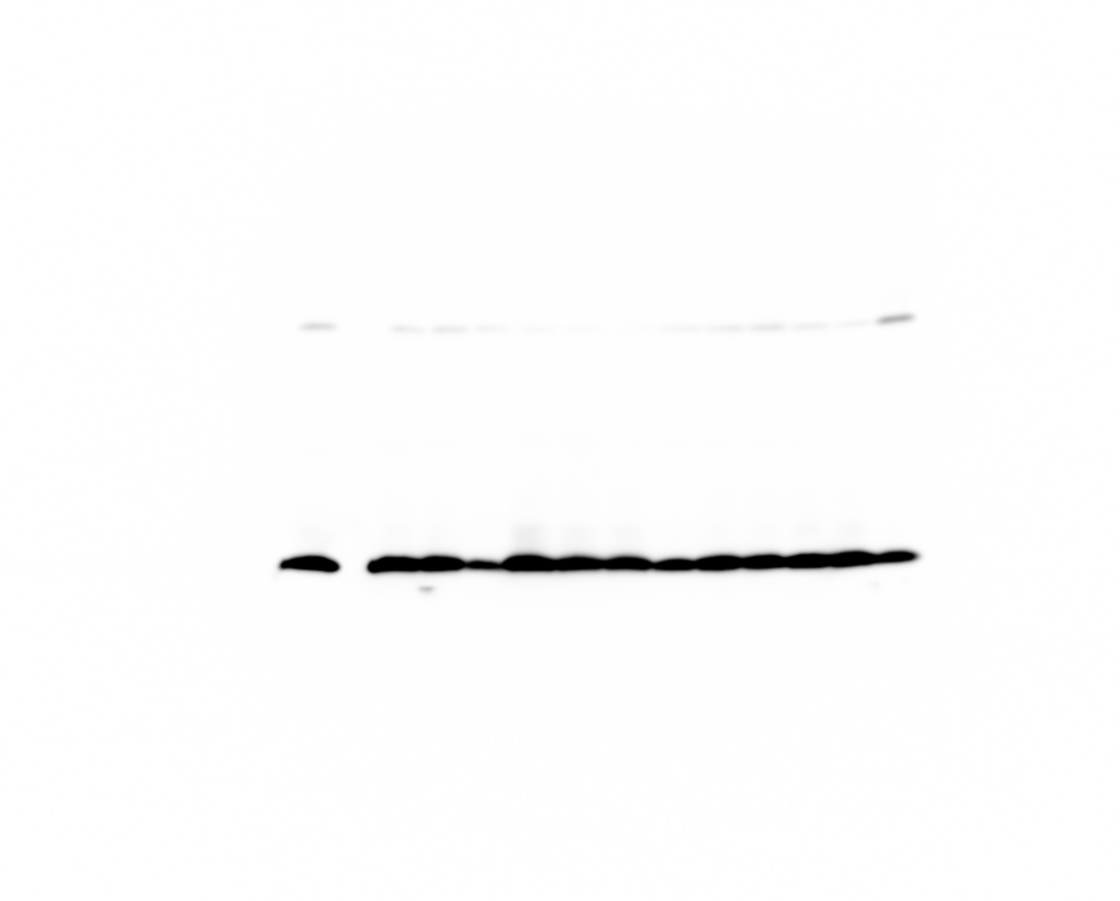

Supplement: Figure 1—source data 1. [file elife-89210-fig1-data1.zip › Figure 1- Source Data 1/individual images/cyto_c_5_min_exposure_gamma_on.jpg]

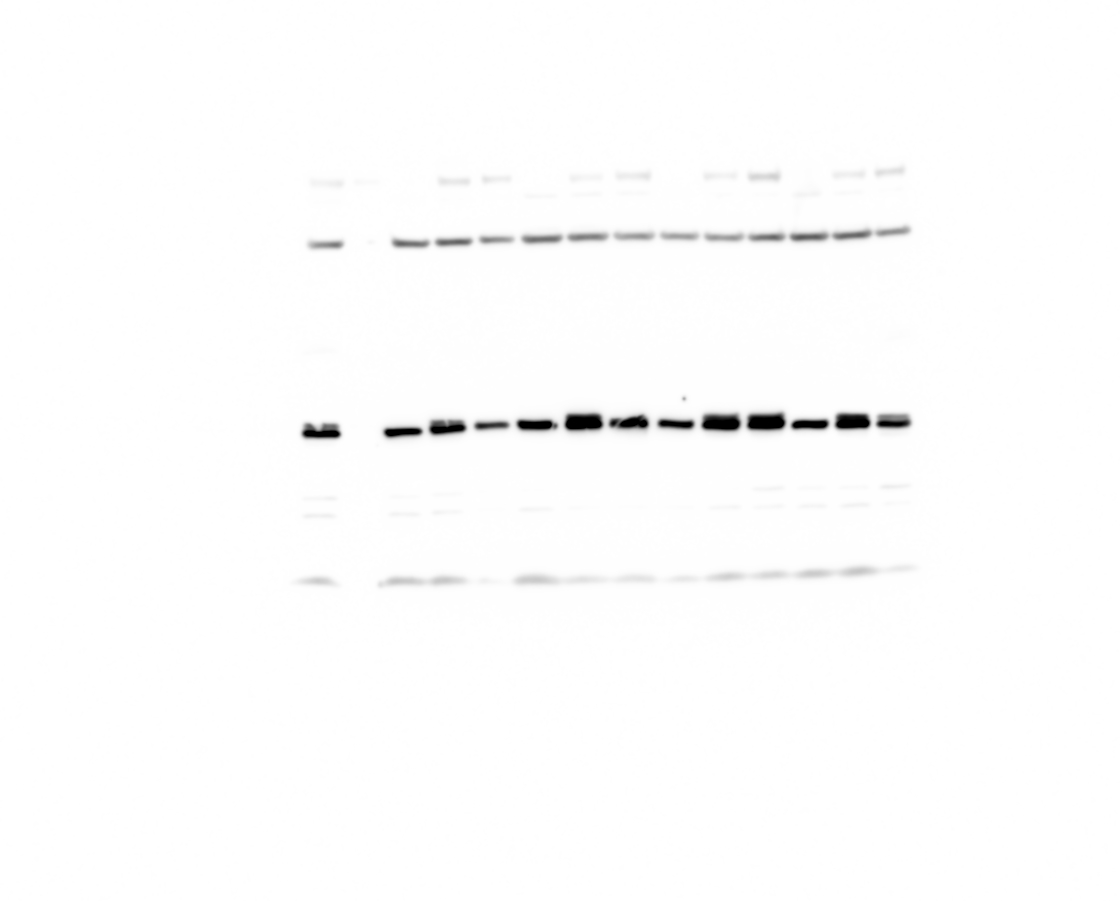

Supplement: Figure 1—source data 1. [file elife-89210-fig1-data1.zip › Figure 1- Source Data 1/individual images/GAPDH_1_min_exposure_gamma_onl.jpg]

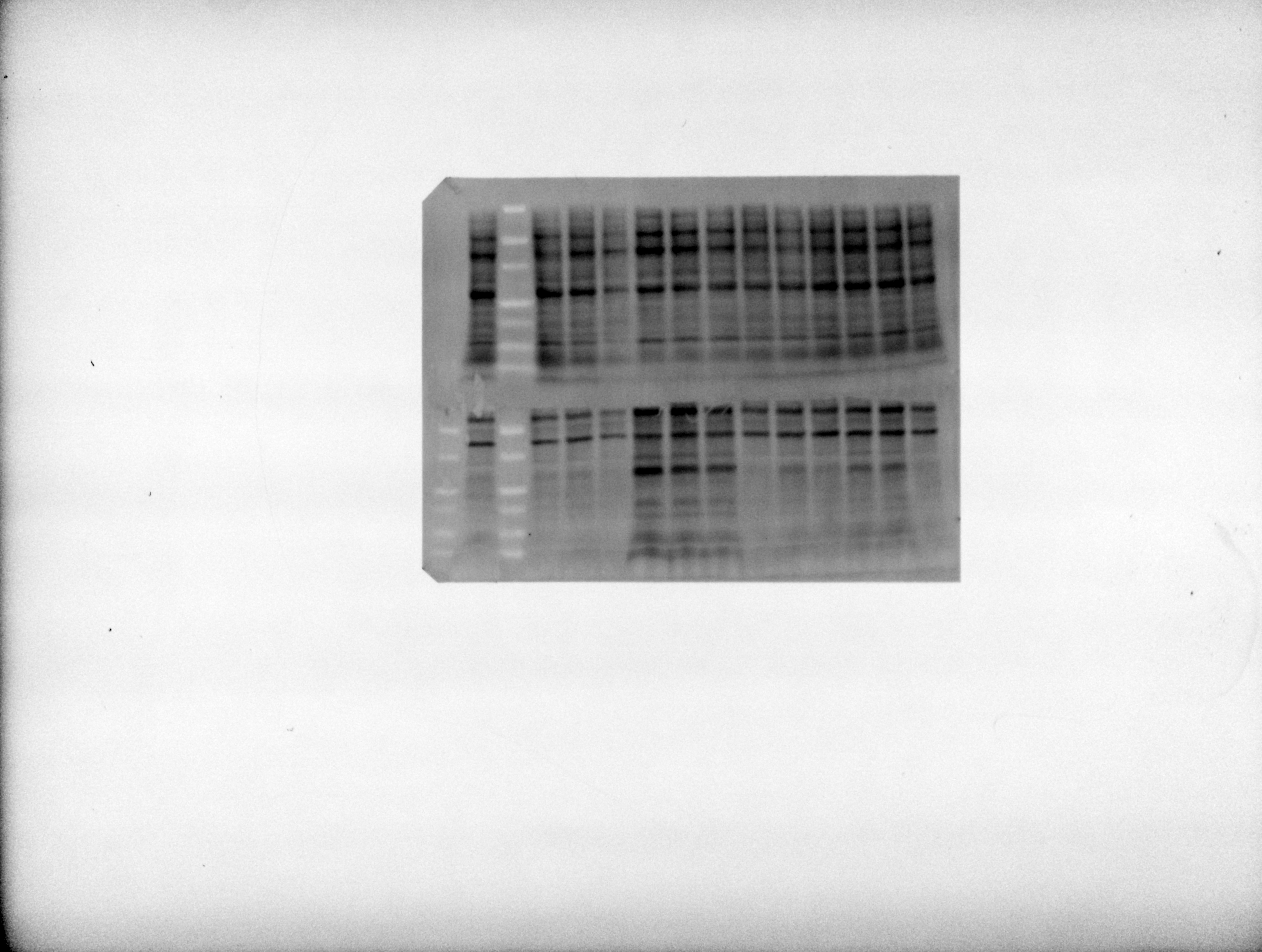

Supplement: Figure 1—source data 1. [file elife-89210-fig1-data1.zip › Figure 1- Source Data 1/individual images/total_protein_membrane.jpg]

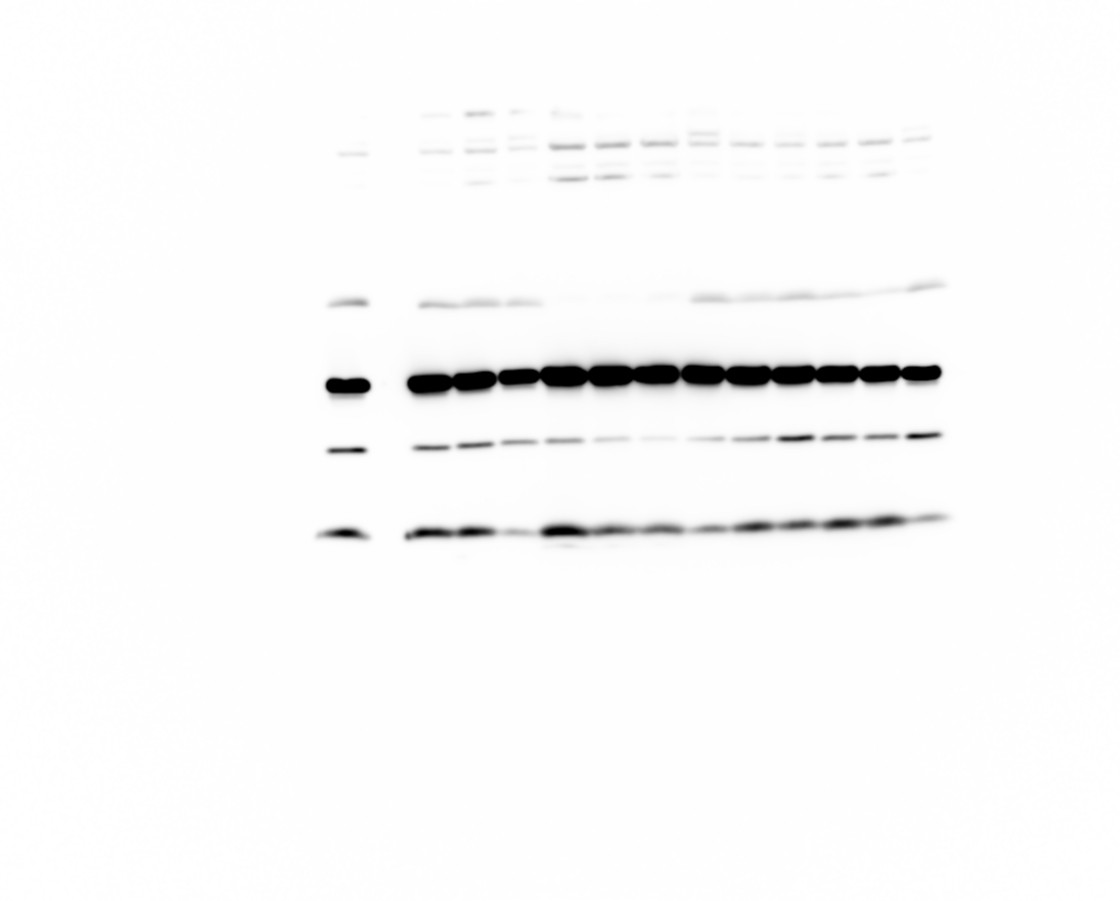

Supplement: Figure 1—source data 1. [file elife-89210-fig1-data1.zip › Figure 1- Source Data 1/individual images/VDAC_5_min_exposure_gamma_only.jpg]

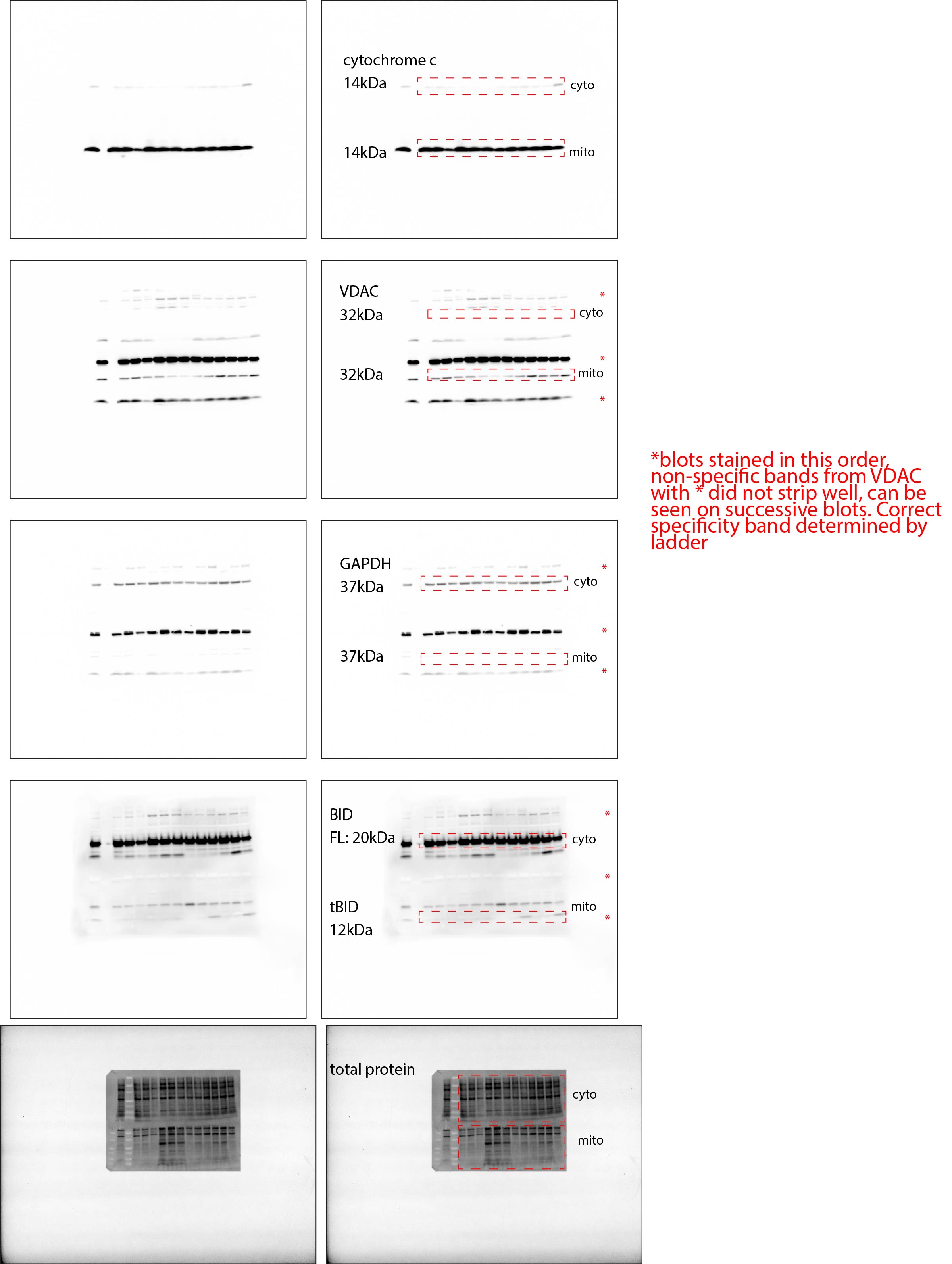

Supplement: Figure 1—source data 1. [file elife-89210-fig1-data1.zip › Figure 1- Source Data 1/western blots.jpg]

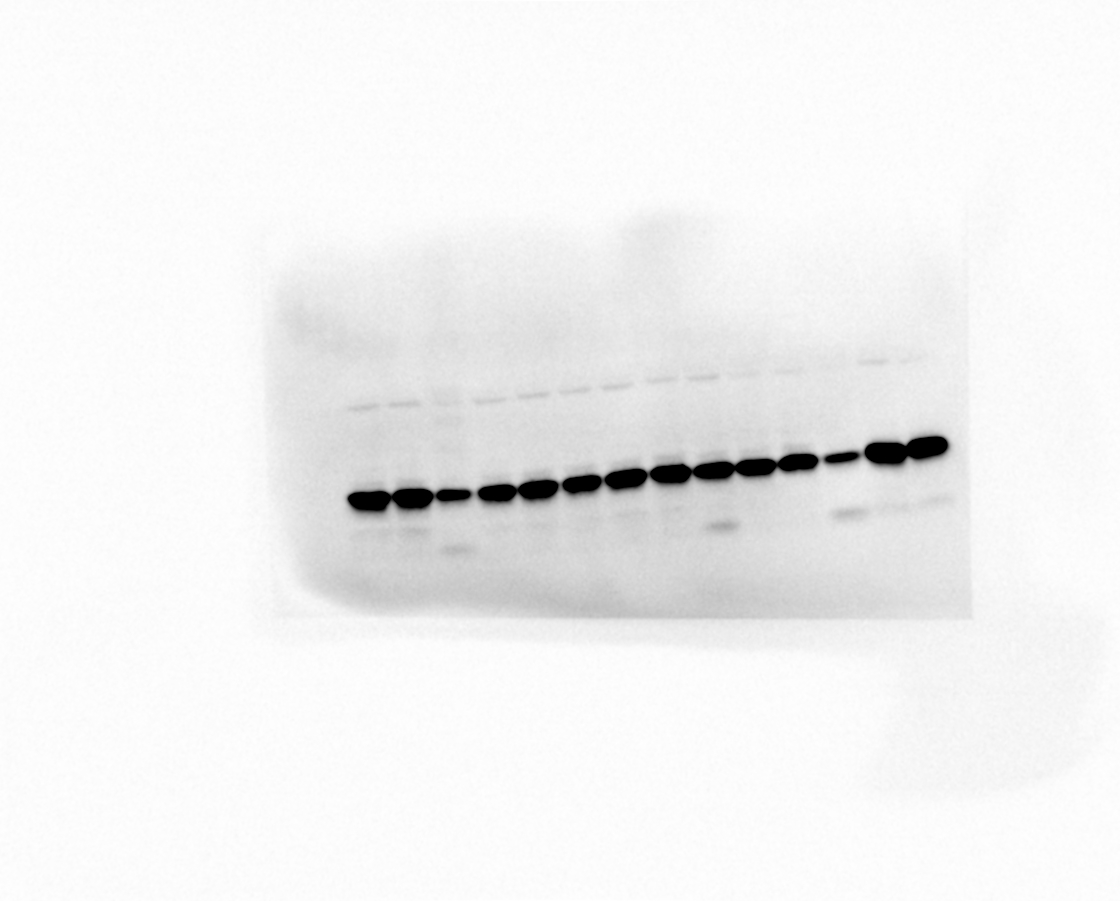

Supplement: Figure 1—source data 2. [file elife-89210-fig1-data2.zip › Figure 1- Source Data 2/individual pictures/4h_BID_10_min_exposure.jpg]

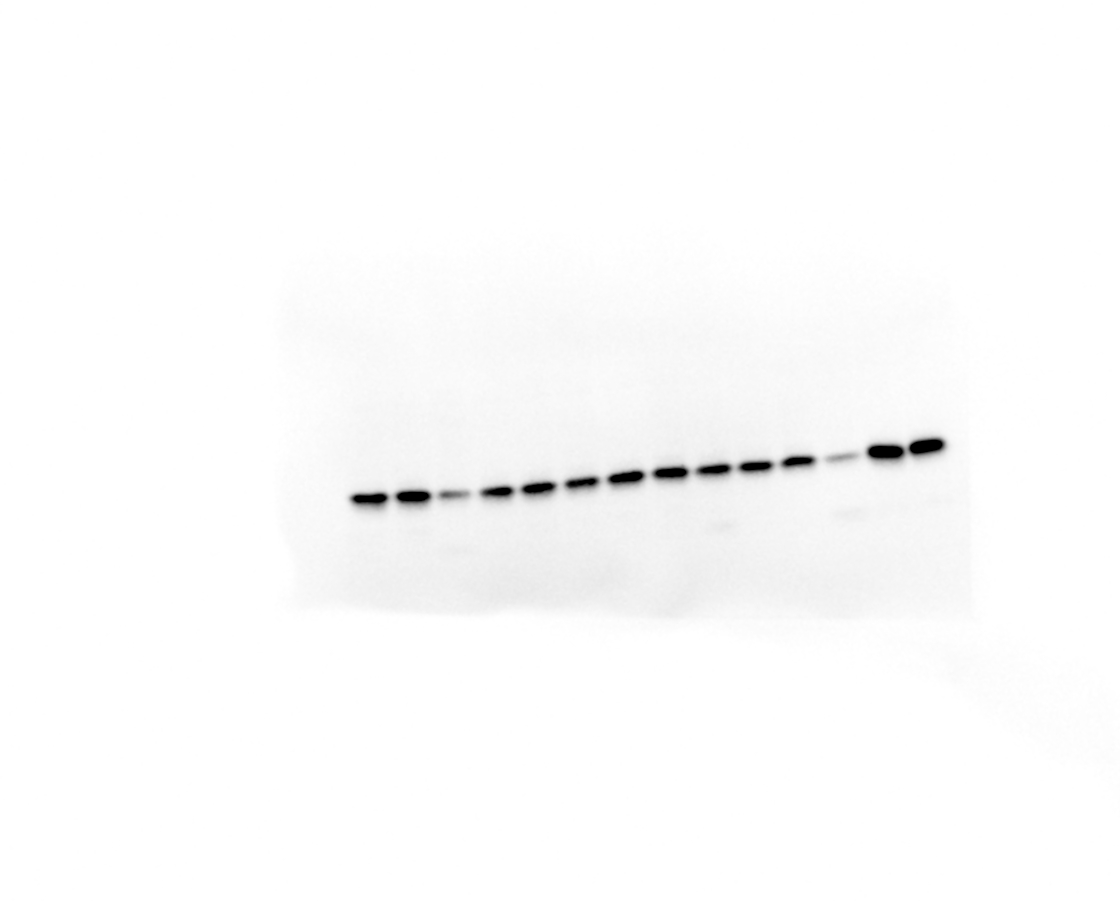

Supplement: Figure 1—source data 2. [file elife-89210-fig1-data2.zip › Figure 1- Source Data 2/individual pictures/4h_BID_2_min_exposure_gamma_on.jpg]

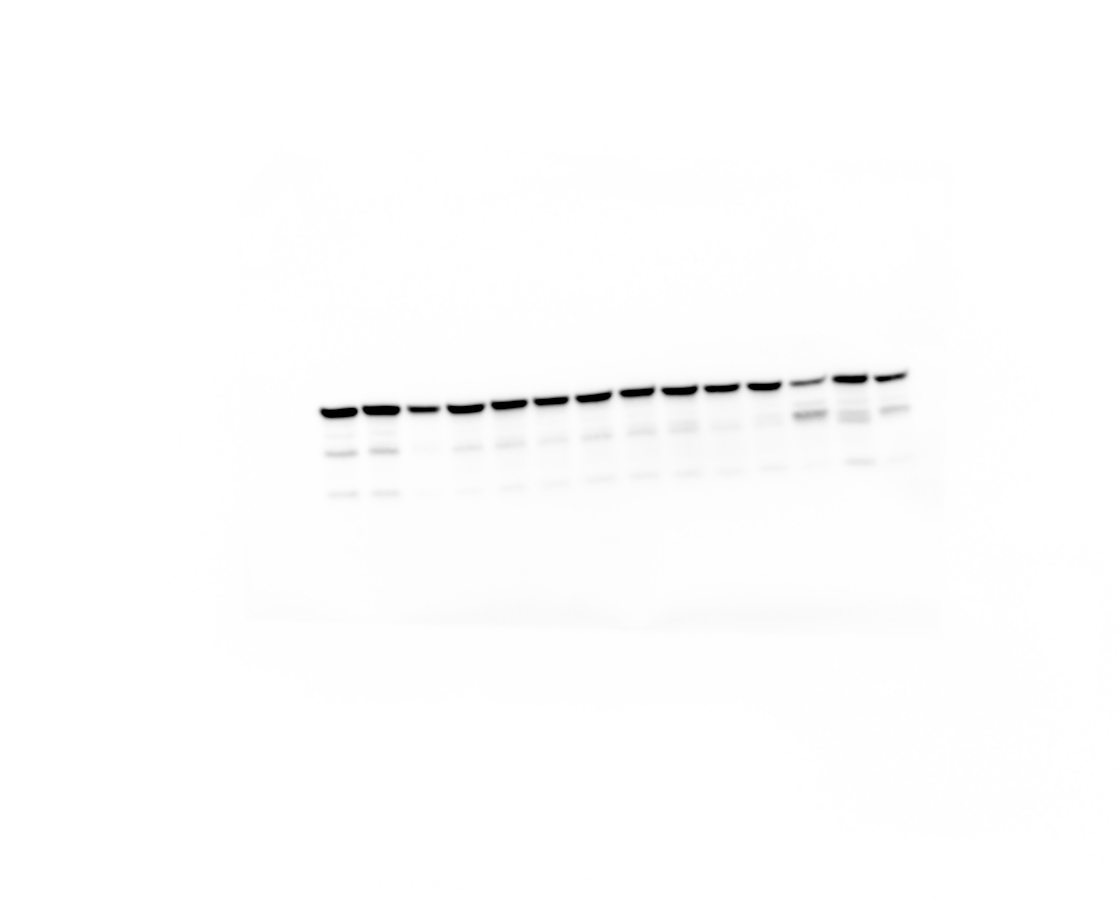

Supplement: Figure 1—source data 2. [file elife-89210-fig1-data2.zip › Figure 1- Source Data 2/individual pictures/4h_casp9_2_min_exposure_gamma_.jpg]

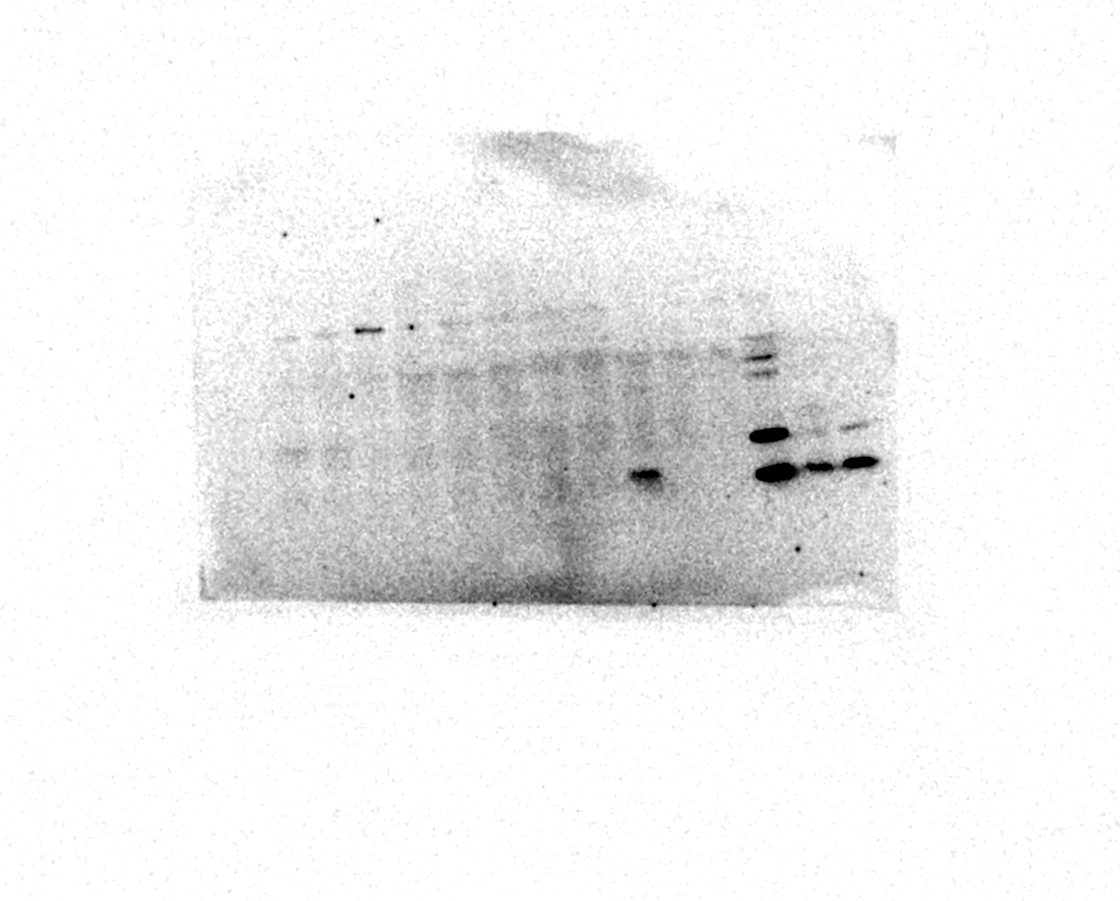

Supplement: Figure 1—source data 2. [file elife-89210-fig1-data2.zip › Figure 1- Source Data 2/individual pictures/4h_ccasp3_5_min_exposure_gamma.jpg]

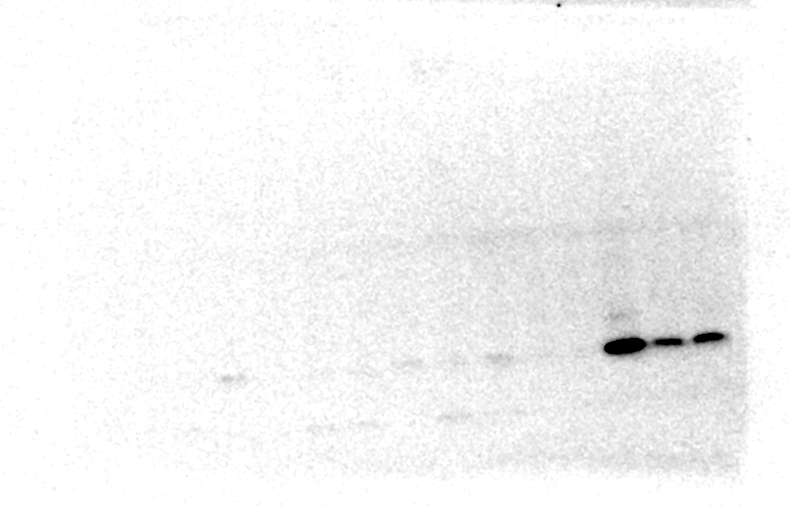

Supplement: Figure 1—source data 2. [file elife-89210-fig1-data2.zip › Figure 1- Source Data 2/individual pictures/4h_ccasp7_2_min_exposure_gamma.jpg]

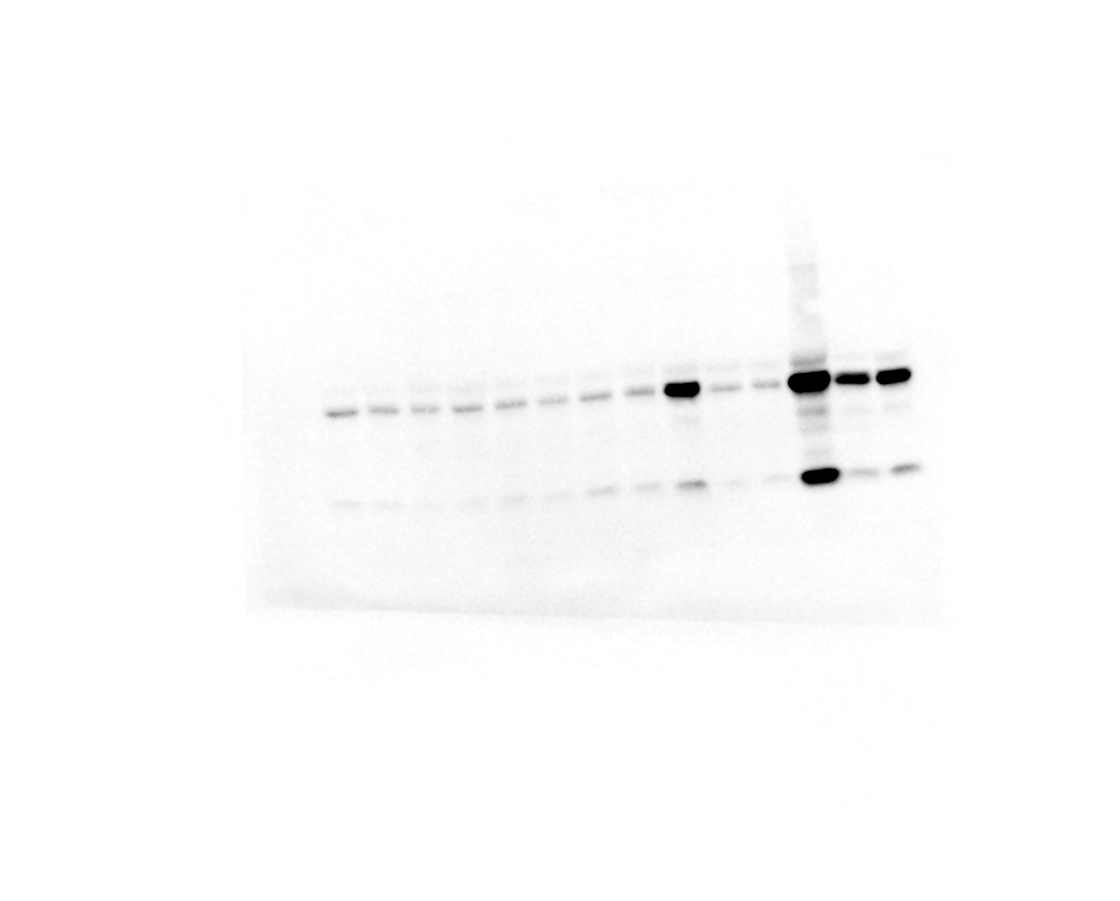

Supplement: Figure 1—source data 2. [file elife-89210-fig1-data2.zip › Figure 1- Source Data 2/individual pictures/4h_ccasp8_2_min_exposure_gamma.jpg]

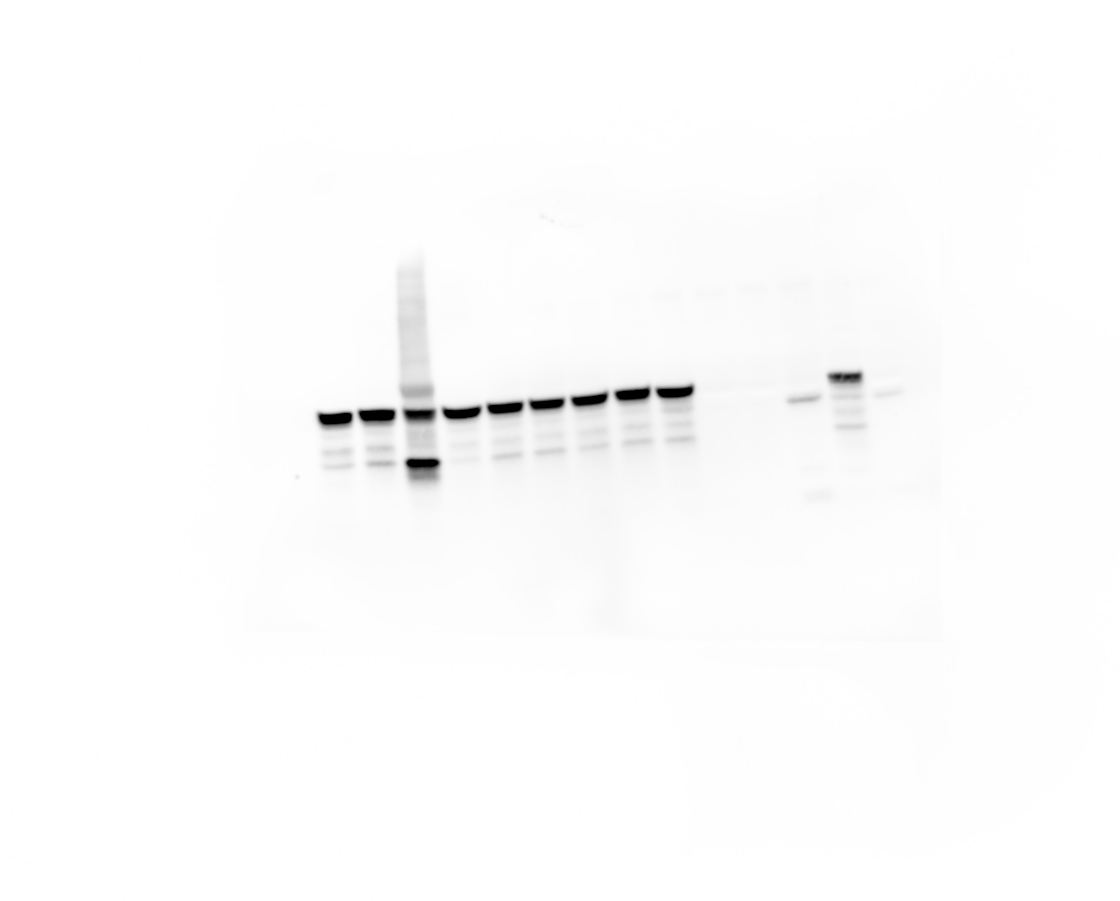

Supplement: Figure 1—source data 2. [file elife-89210-fig1-data2.zip › Figure 1- Source Data 2/individual pictures/4h_gsdmD_11_min_exposure_gamma.jpg]

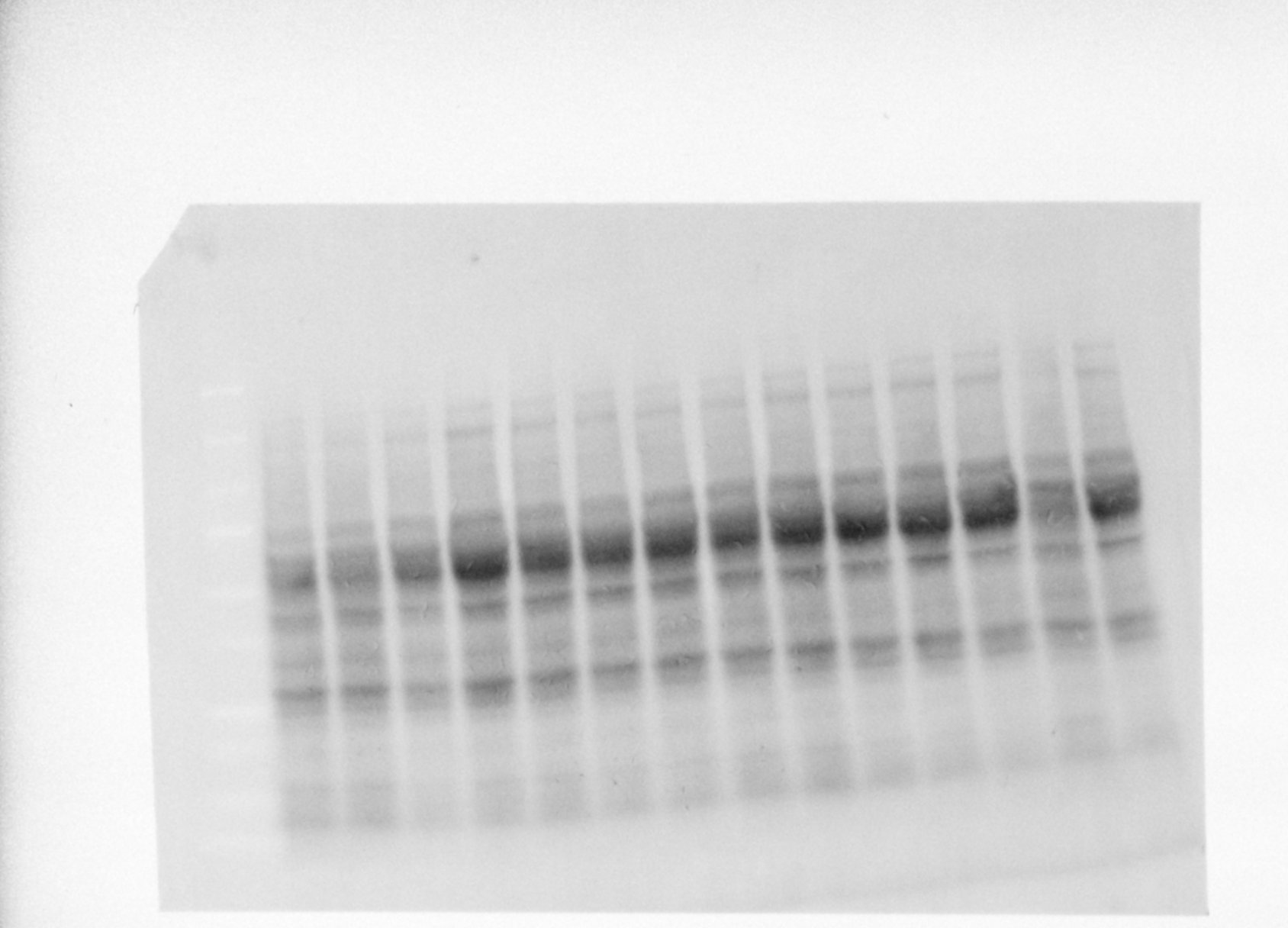

Supplement: Figure 1—source data 2. [file elife-89210-fig1-data2.zip › Figure 1- Source Data 2/individual pictures/total_protein_membrane_4h_.jpg]

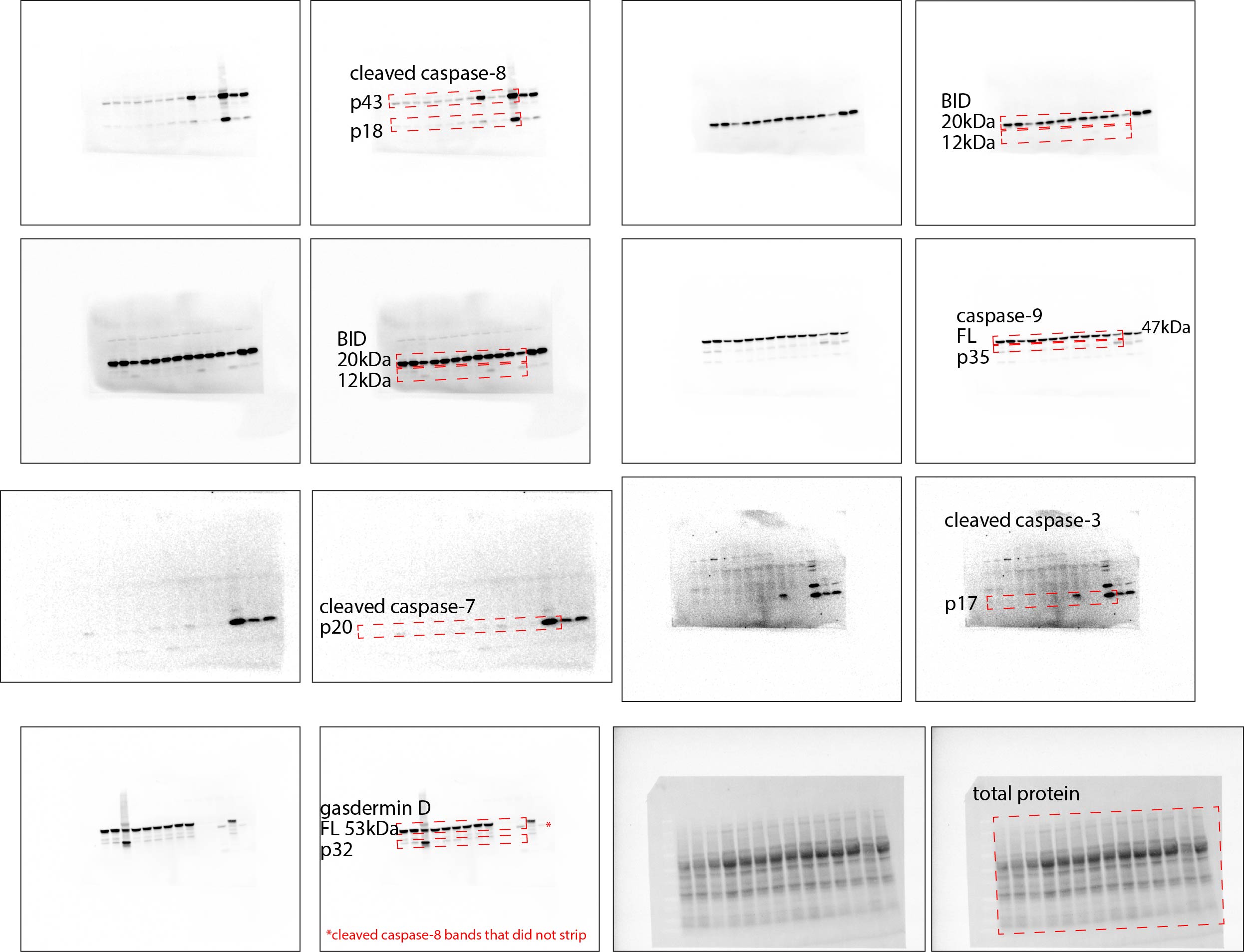

Supplement: Figure 1—source data 2. [file elife-89210-fig1-data2.zip › Figure 1- Source Data 2/western blots.jpg]

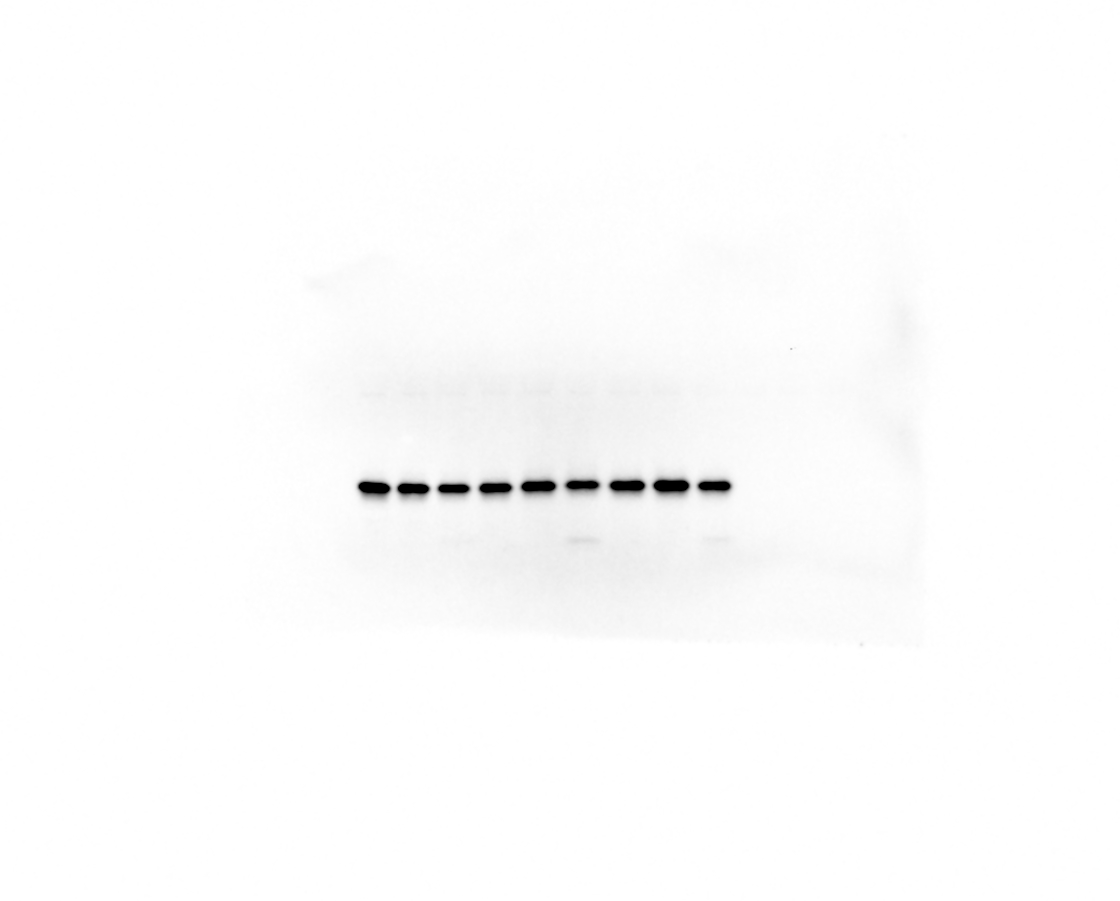

Supplement: Figure 1—figure supplement 1—source data 1. [file elife-89210-fig1-figsupp1-data1.zip › Figure 1- figure supplement 1- Source Data 1/individual images/BID_5_.jpg]

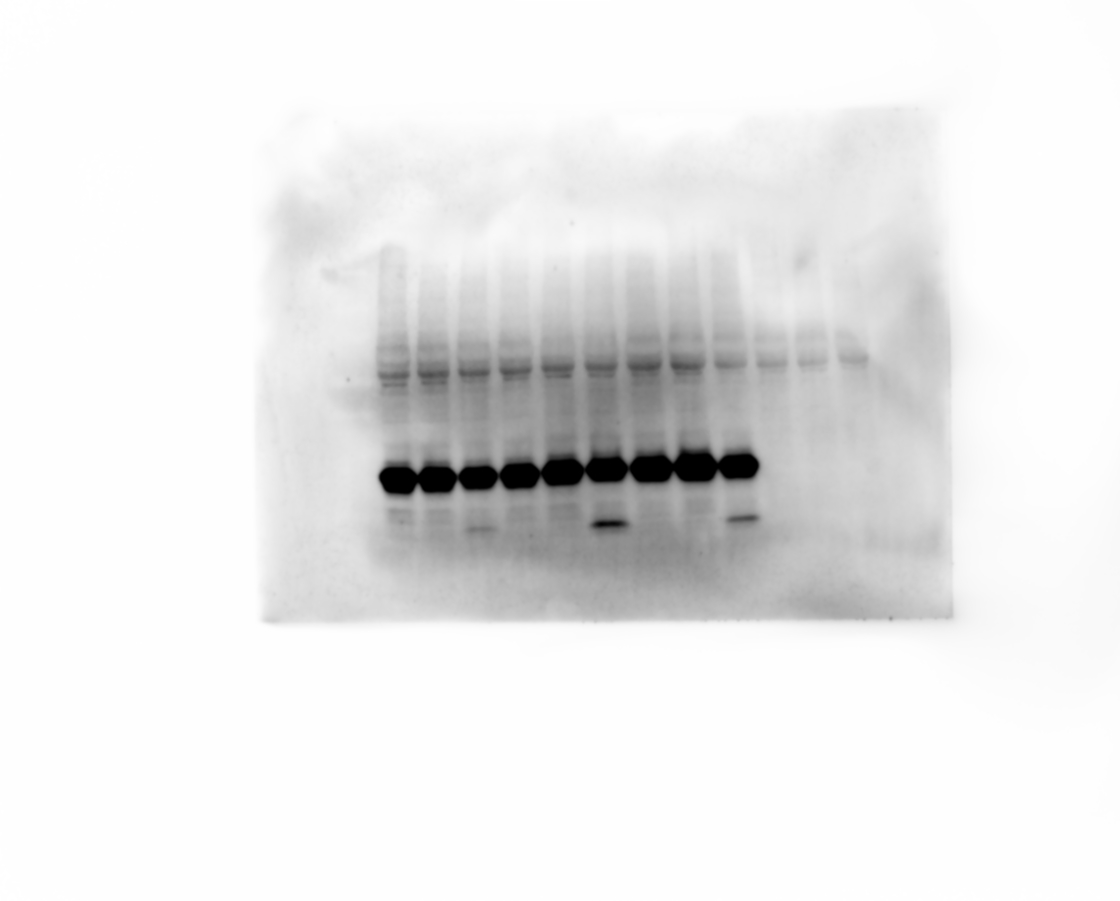

Supplement: Figure 1—figure supplement 1—source data 1. [file elife-89210-fig1-figsupp1-data1.zip › Figure 1- figure supplement 1- Source Data 1/individual images/BID_fe.jpg]

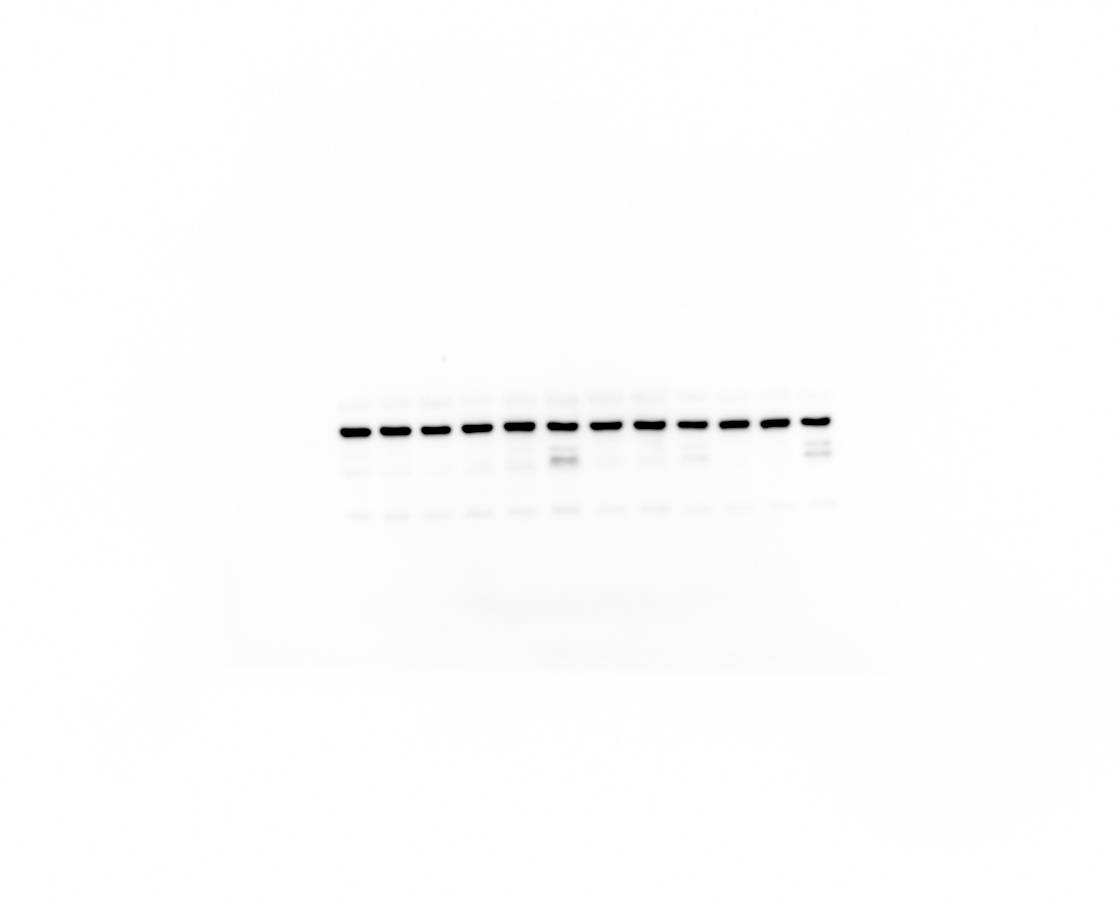

Supplement: Figure 1—figure supplement 1—source data 1. [file elife-89210-fig1-figsupp1-data1.zip › Figure 1- figure supplement 1- Source Data 1/individual images/casp9_.jpg]

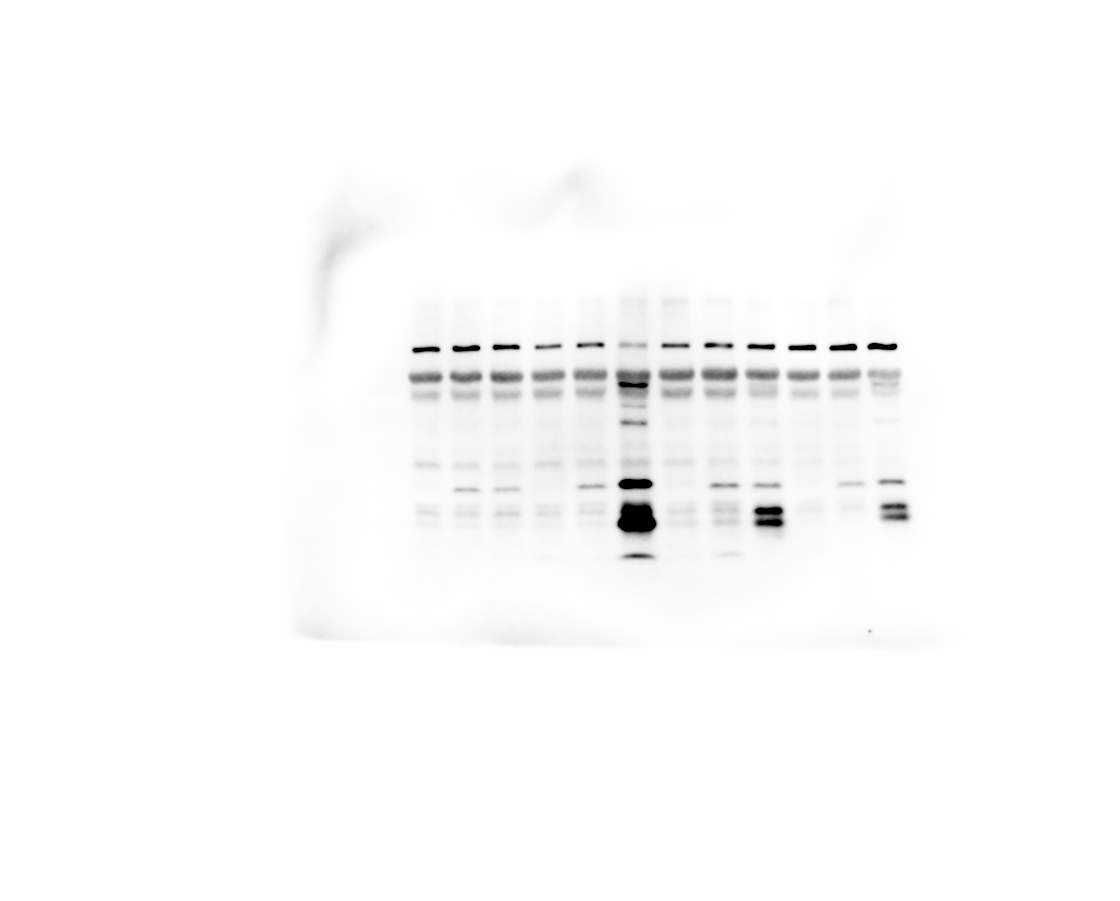

Supplement: Figure 1—figure supplement 1—source data 1. [file elife-89210-fig1-figsupp1-data1.zip › Figure 1- figure supplement 1- Source Data 1/individual images/ccasp3.jpg]

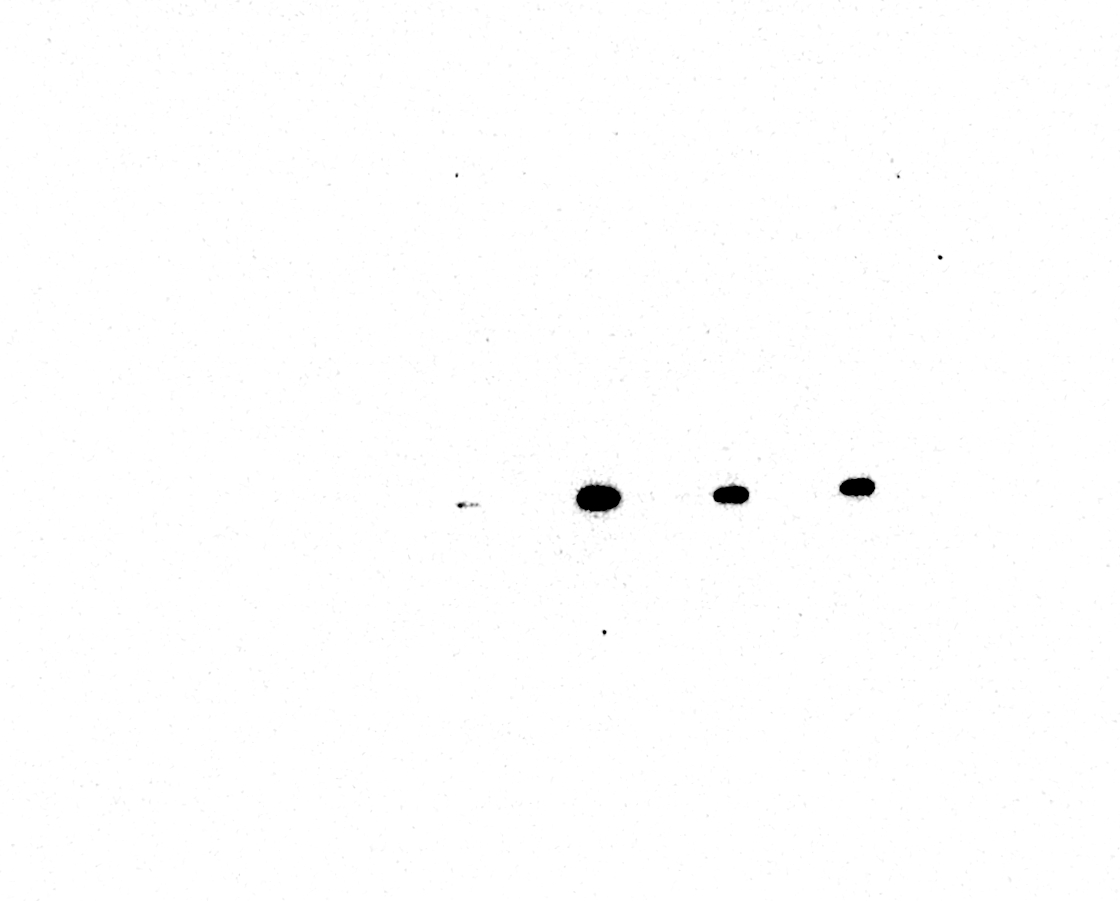

Supplement: Figure 1—figure supplement 1—source data 1. [file elife-89210-fig1-figsupp1-data1.zip › Figure 1- figure supplement 1- Source Data 1/individual images/ccasp7.jpg]

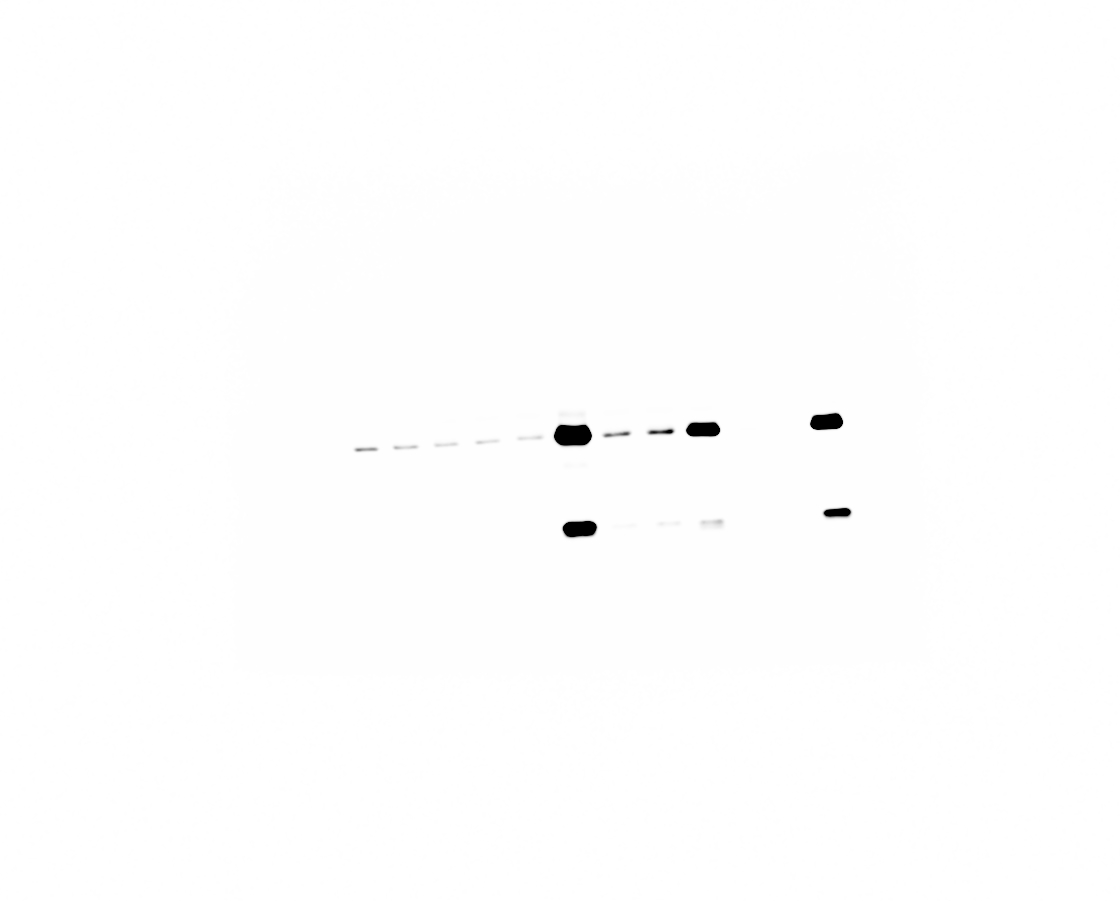

Supplement: Figure 1—figure supplement 1—source data 1. [file elife-89210-fig1-figsupp1-data1.zip › Figure 1- figure supplement 1- Source Data 1/individual images/ccasp8.jpg]

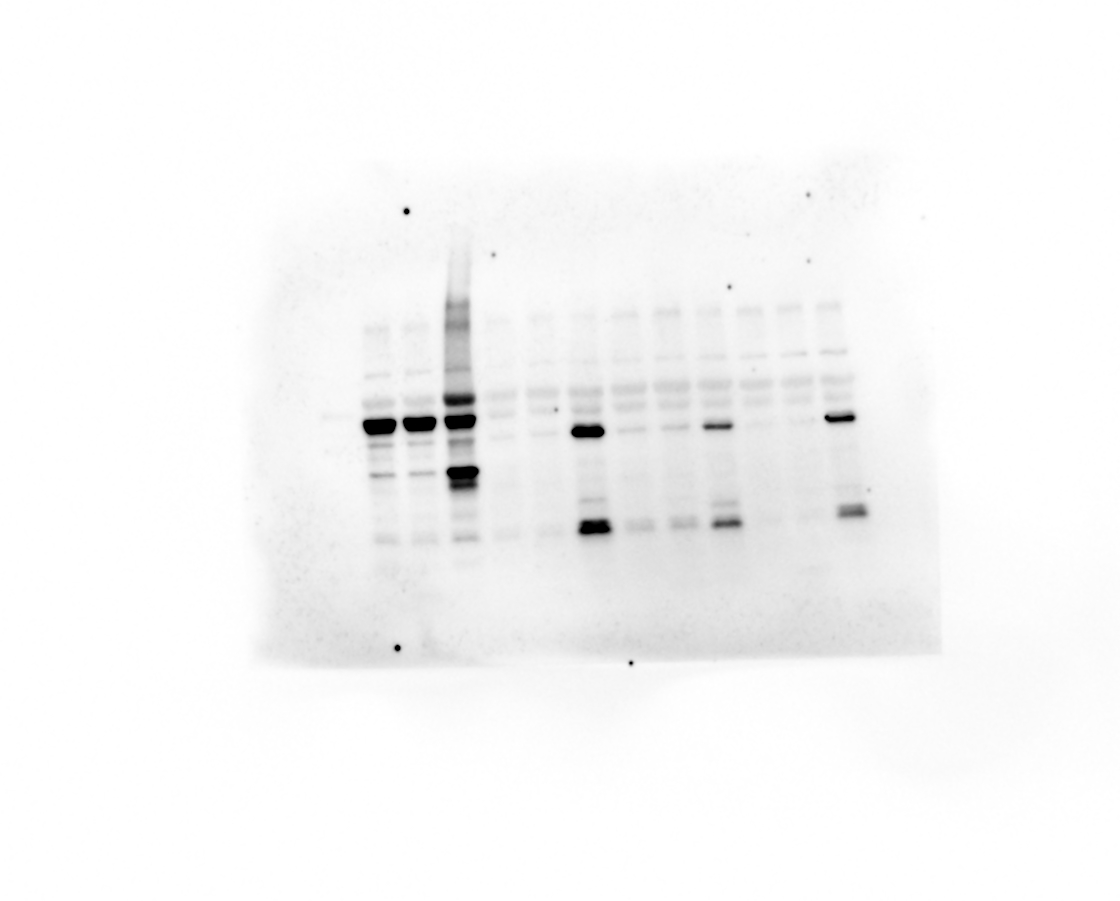

Supplement: Figure 1—figure supplement 1—source data 1. [file elife-89210-fig1-figsupp1-data1.zip › Figure 1- figure supplement 1- Source Data 1/individual images/gsdmD_.jpg]

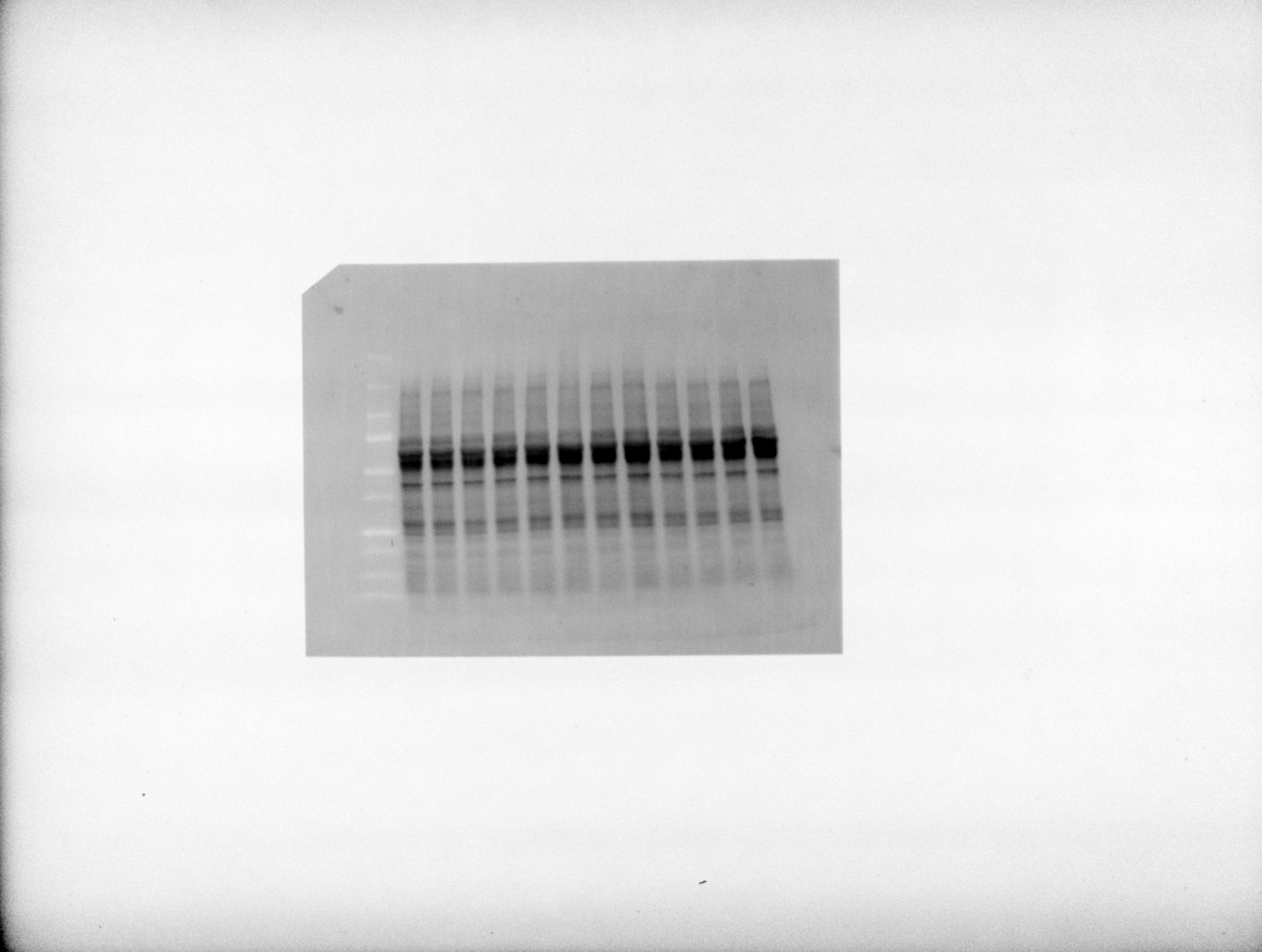

Supplement: Figure 1—figure supplement 1—source data 1. [file elife-89210-fig1-figsupp1-data1.zip › Figure 1- figure supplement 1- Source Data 1/individual images/total_.jpg]

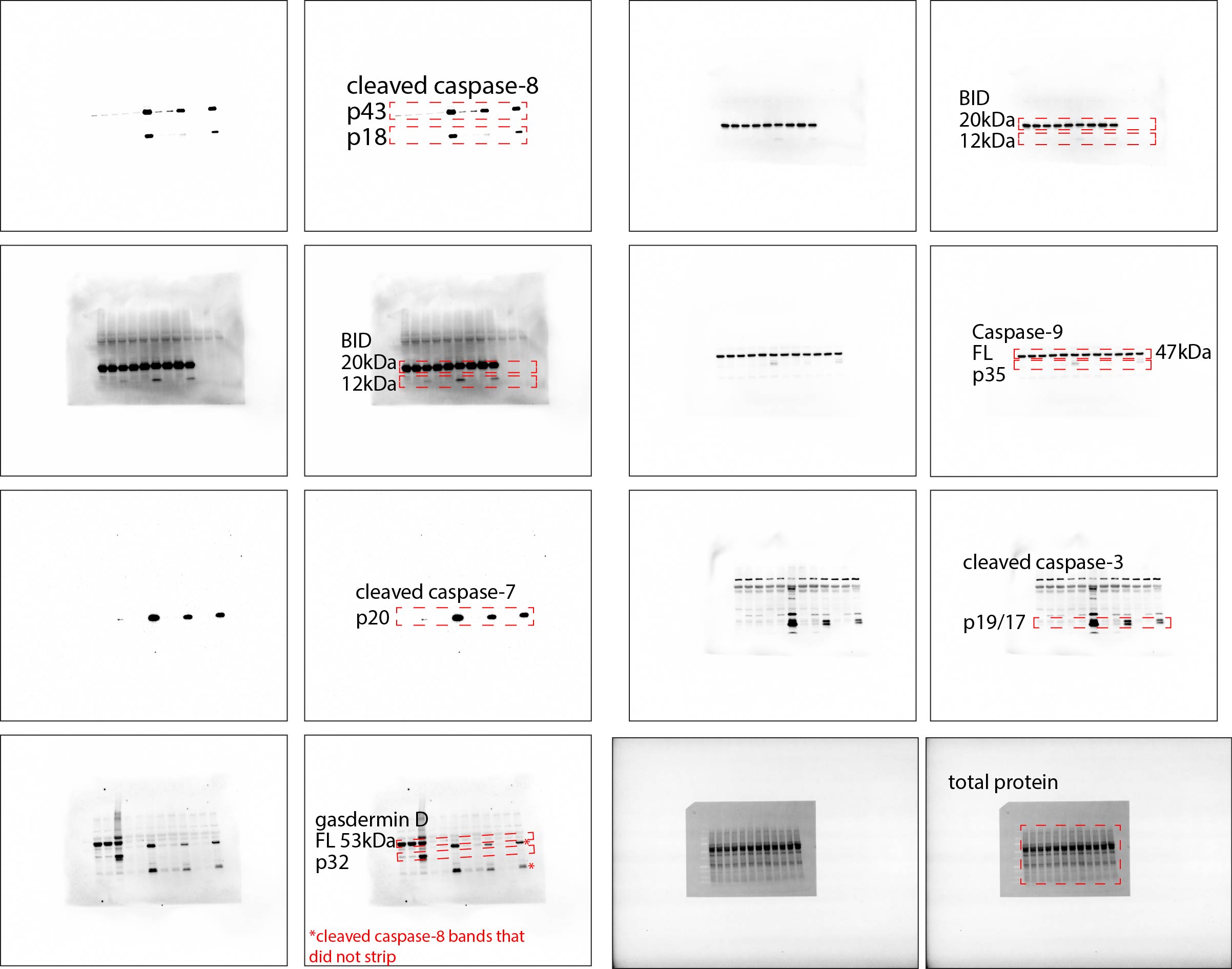

Supplement: Figure 1—figure supplement 1—source data 1. [file elife-89210-fig1-figsupp1-data1.zip › Figure 1- figure supplement 1- Source Data 1/western blots.jpg]

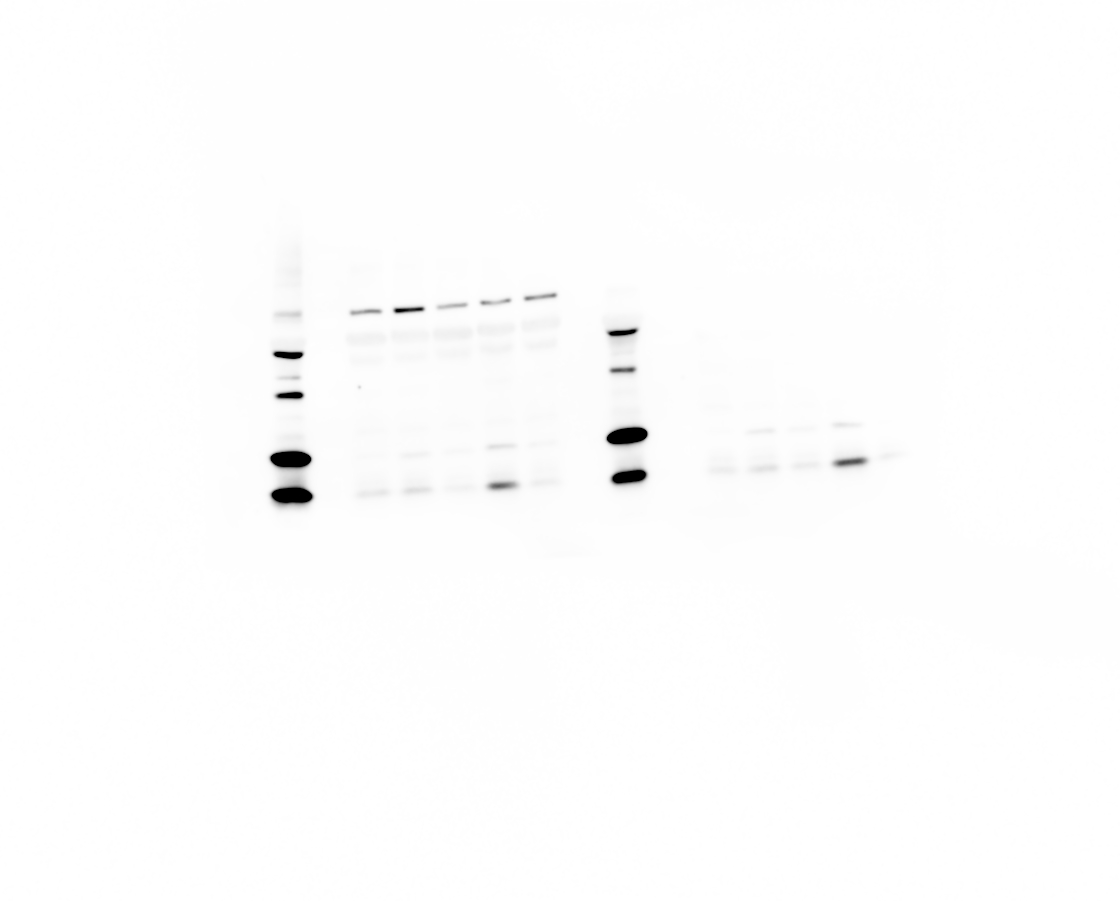

Supplement: Figure 3—source data 1. [file elife-89210-fig3-data1.zip › Figure 3- Source Data 1/individual pictures/ccasp3_5_min_exposure_femto_ga.jpg]

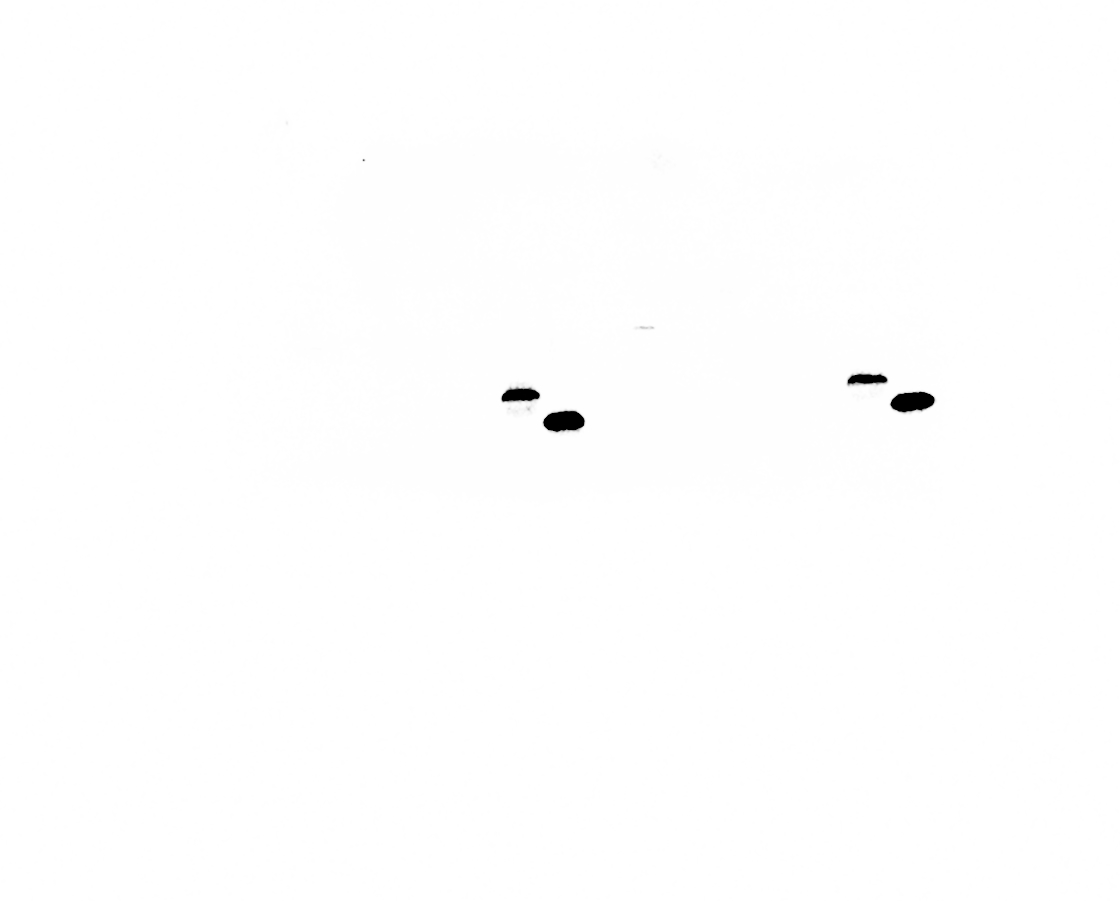

Supplement: Figure 3—source data 1. [file elife-89210-fig3-data1.zip › Figure 3- Source Data 1/individual pictures/HA_1_min_exposure_gamma_only.jpg]

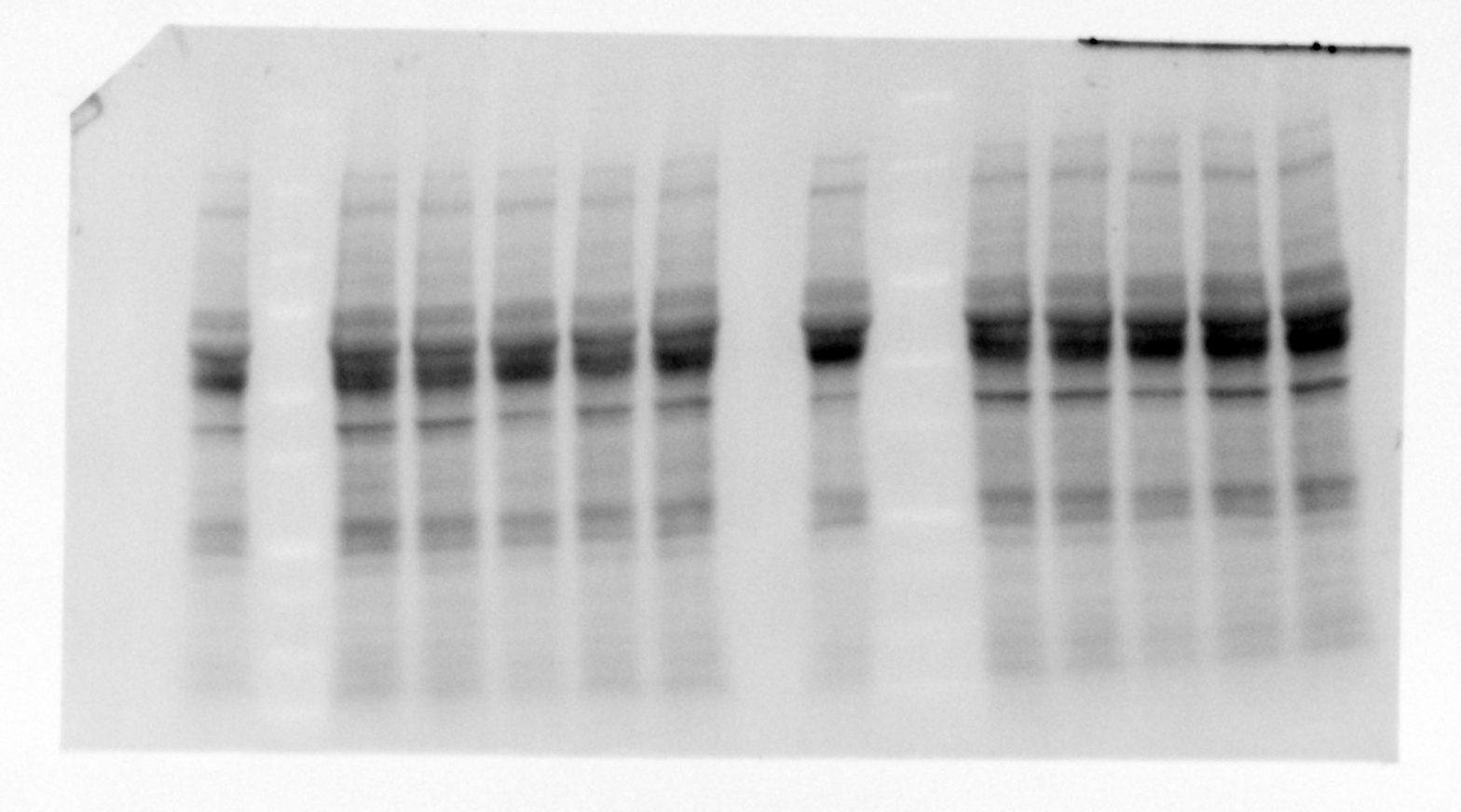

Supplement: Figure 3—source data 1. [file elife-89210-fig3-data1.zip › Figure 3- Source Data 1/individual pictures/total_protein_membrane_invert.jpg]

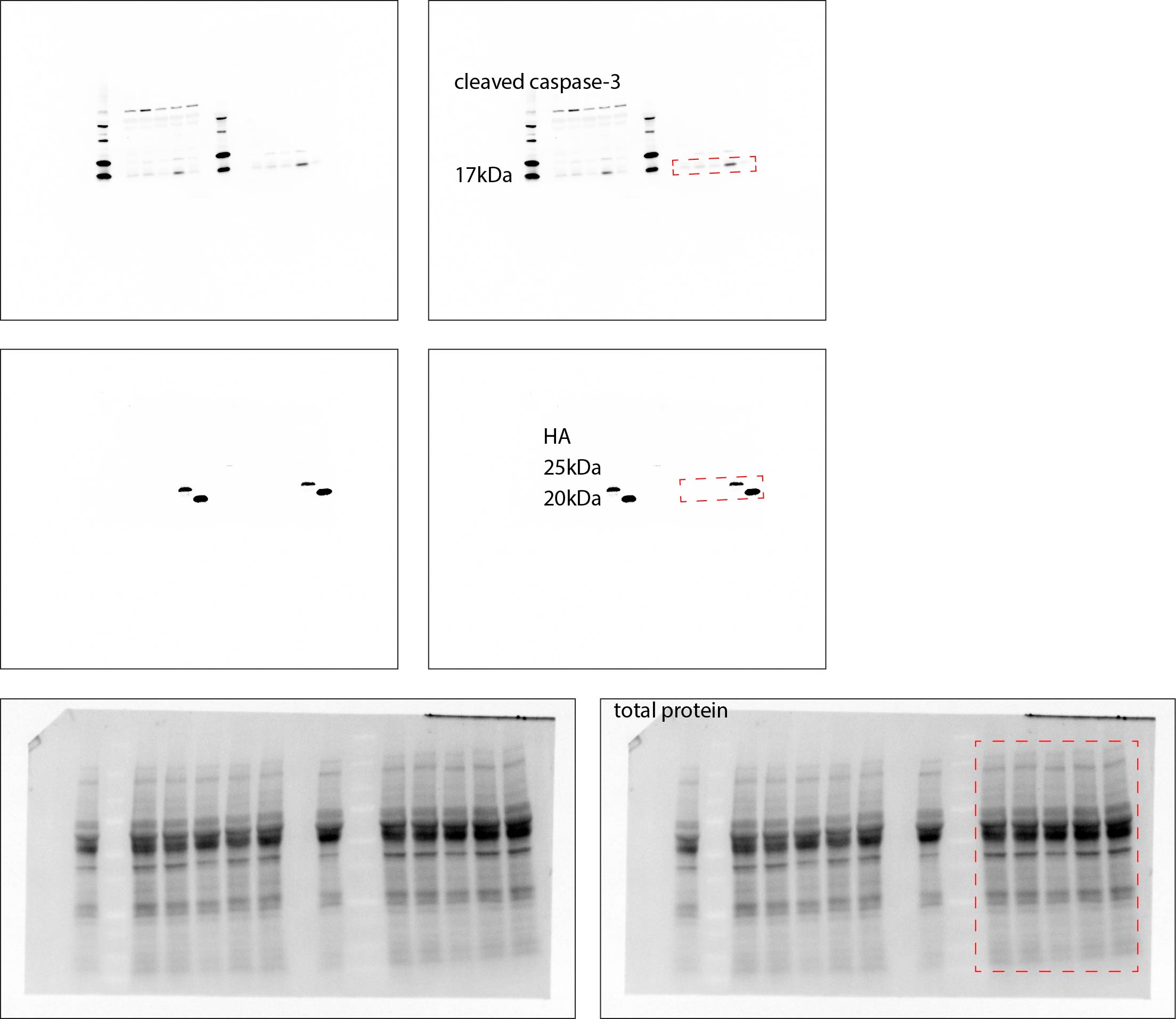

Supplement: Figure 3—source data 1. [file elife-89210-fig3-data1.zip › Figure 3- Source Data 1/western blots.jpg]

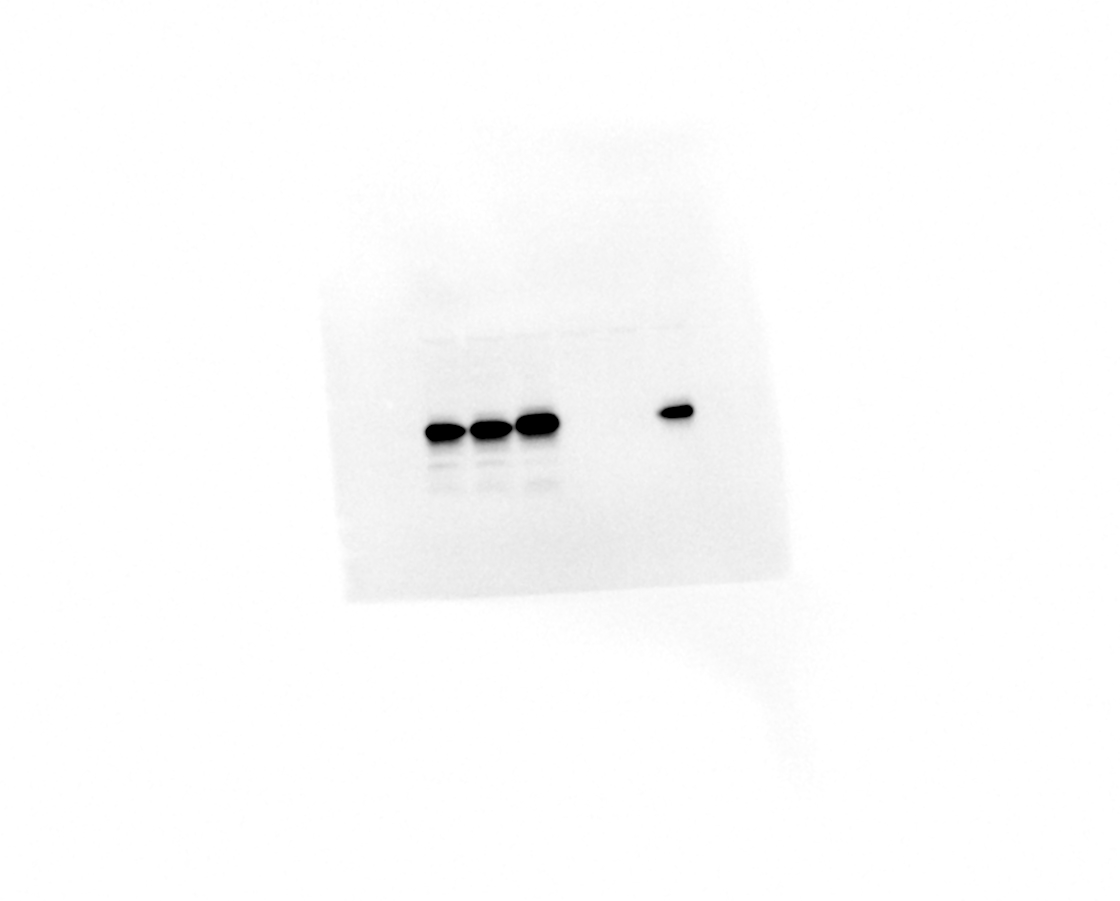

Supplement: Figure 3—source data 2. [file elife-89210-fig3-data2.zip › Figure 3- Source Data 2/individual pics/BID_2_min_exposure_gamma_only.jpg]

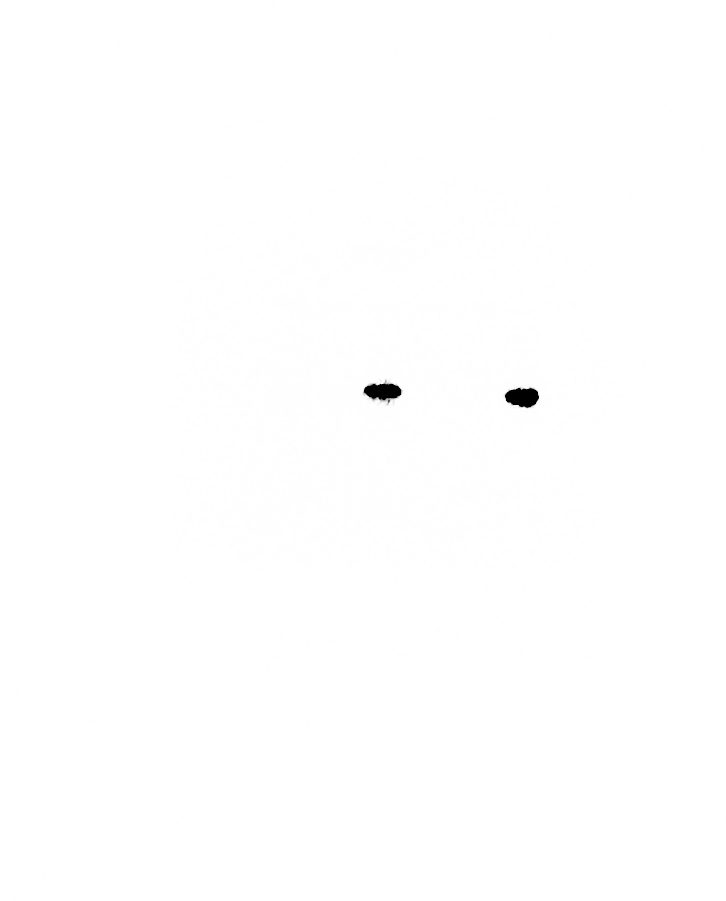

Supplement: Figure 3—source data 2. [file elife-89210-fig3-data2.zip › Figure 3- Source Data 2/individual pics/HA_10s_exposure_gamma_only.jpg]

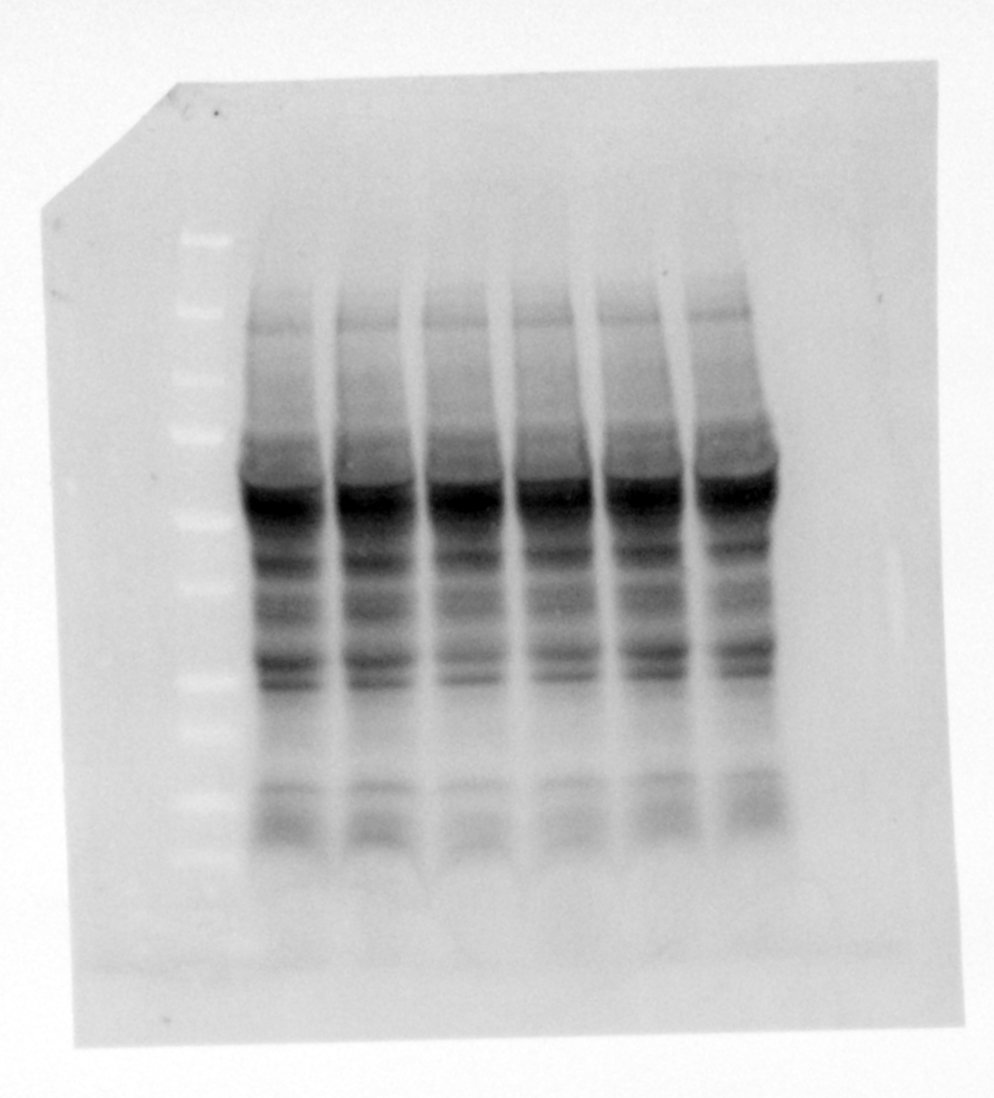

Supplement: Figure 3—source data 2. [file elife-89210-fig3-data2.zip › Figure 3- Source Data 2/individual pics/membrane_total_protein_stain_i.jpg]

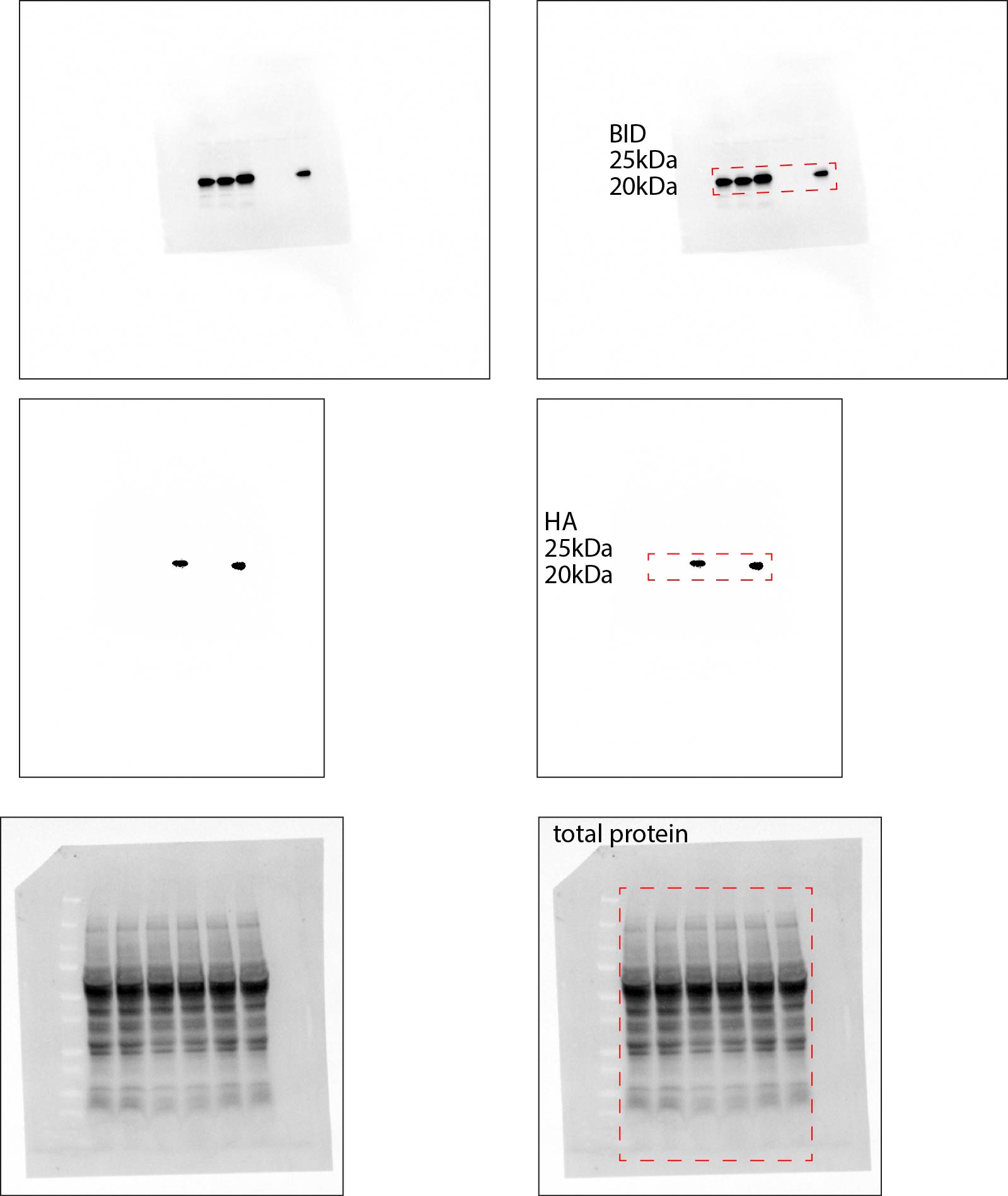

Supplement: Figure 3—source data 2. [file elife-89210-fig3-data2.zip › Figure 3- Source Data 2/western blots.jpg]

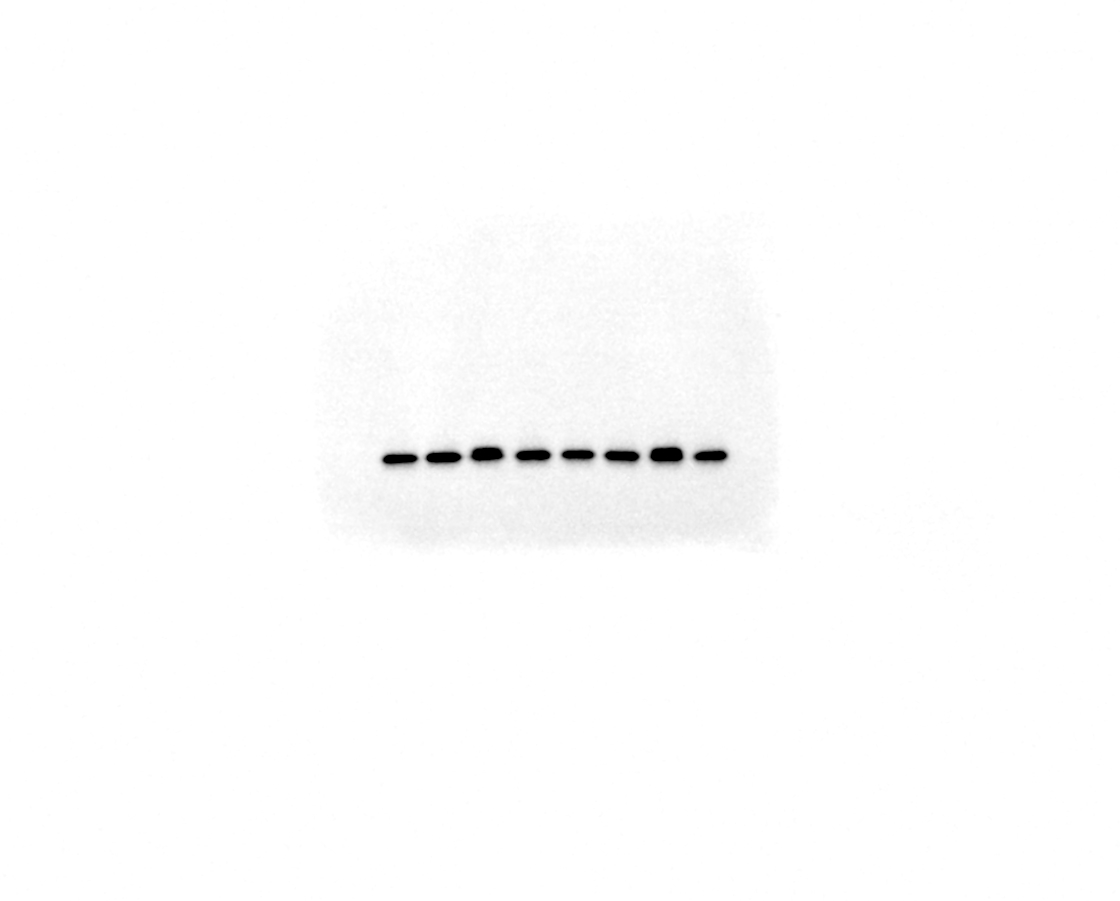

Supplement: Figure 3—source data 3. [file elife-89210-fig3-data3.zip › Figure 3- Source Data 3/individual pictures/BID_1_min_exposure_gamma_only.jpg]

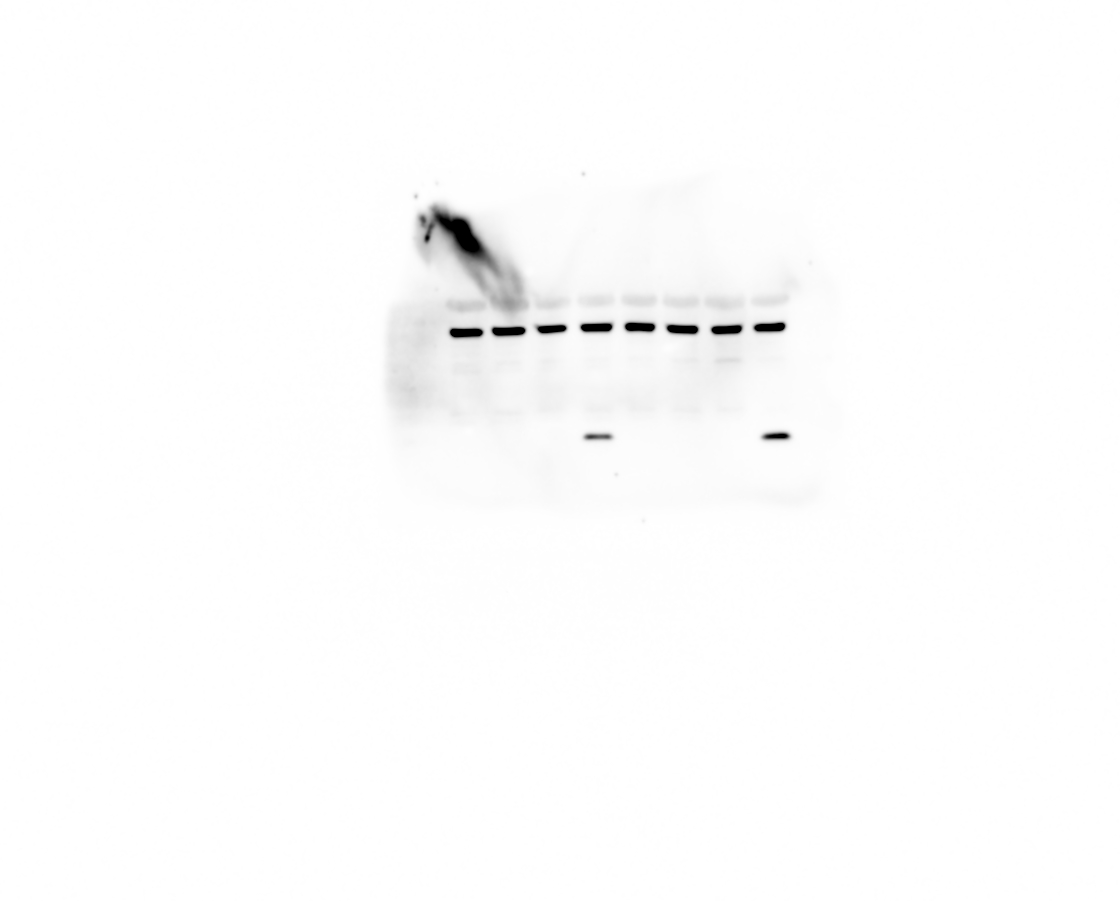

Supplement: Figure 3—source data 3. [file elife-89210-fig3-data3.zip › Figure 3- Source Data 3/individual pictures/casp9_redo_10_min_exposure_gam.jpg]

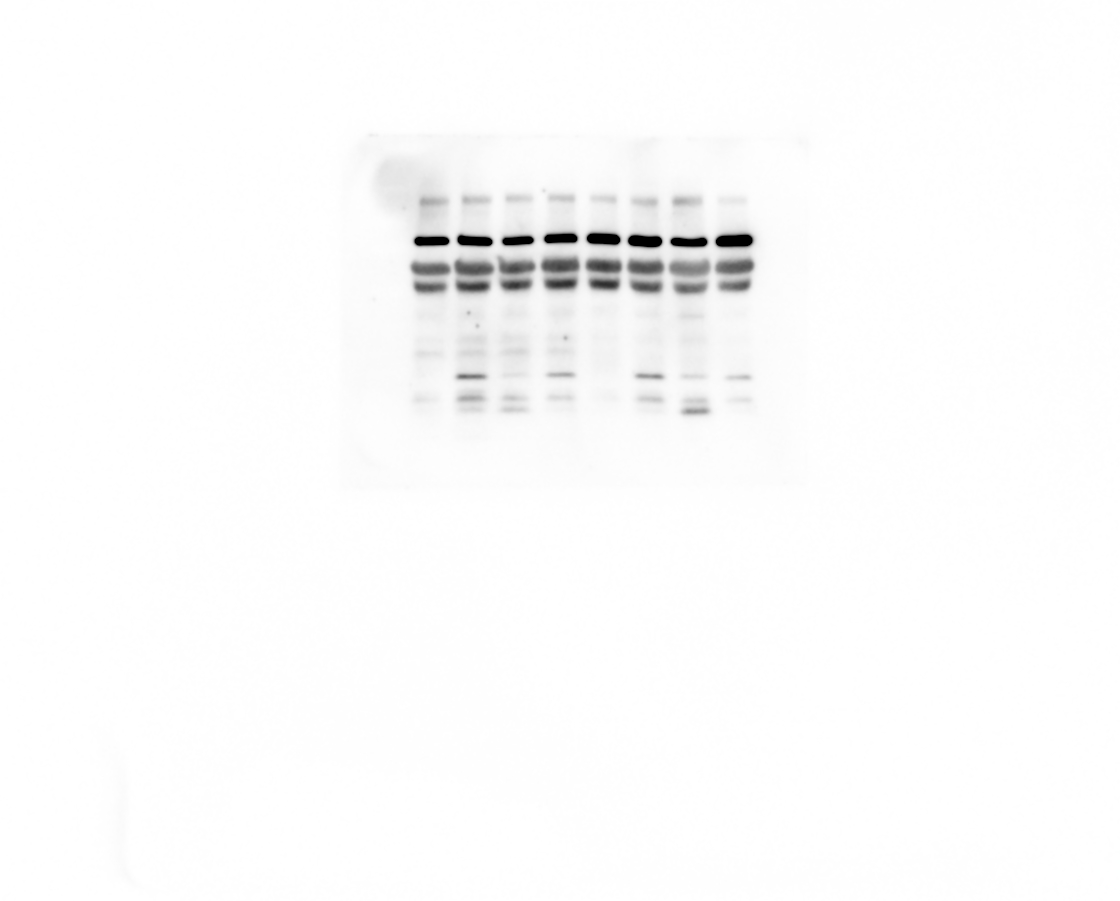

Supplement: Figure 3—source data 3. [file elife-89210-fig3-data3.zip › Figure 3- Source Data 3/individual pictures/ccasp3_10_min_exposure_femto_g.jpg]

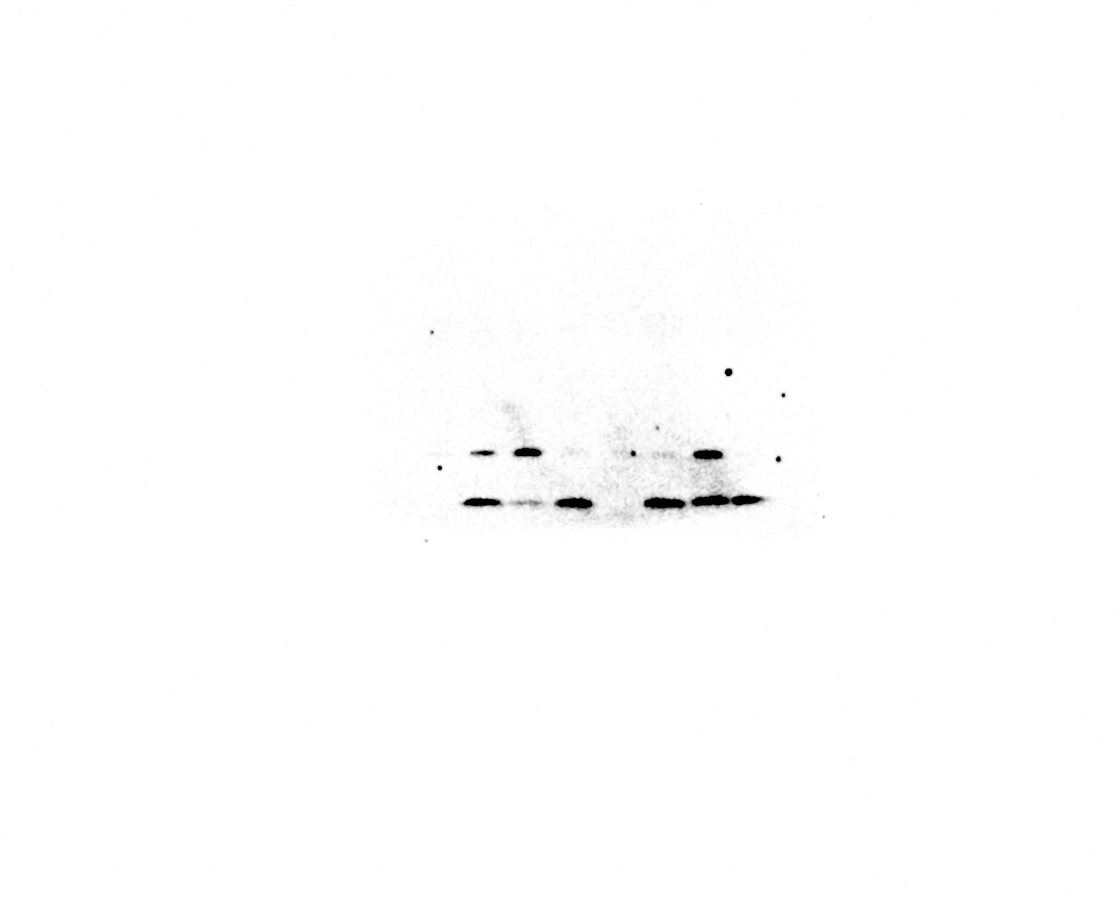

Supplement: Figure 3—source data 3. [file elife-89210-fig3-data3.zip › Figure 3- Source Data 3/individual pictures/ccasp7_5_min_exposure_gamma_on.jpg]

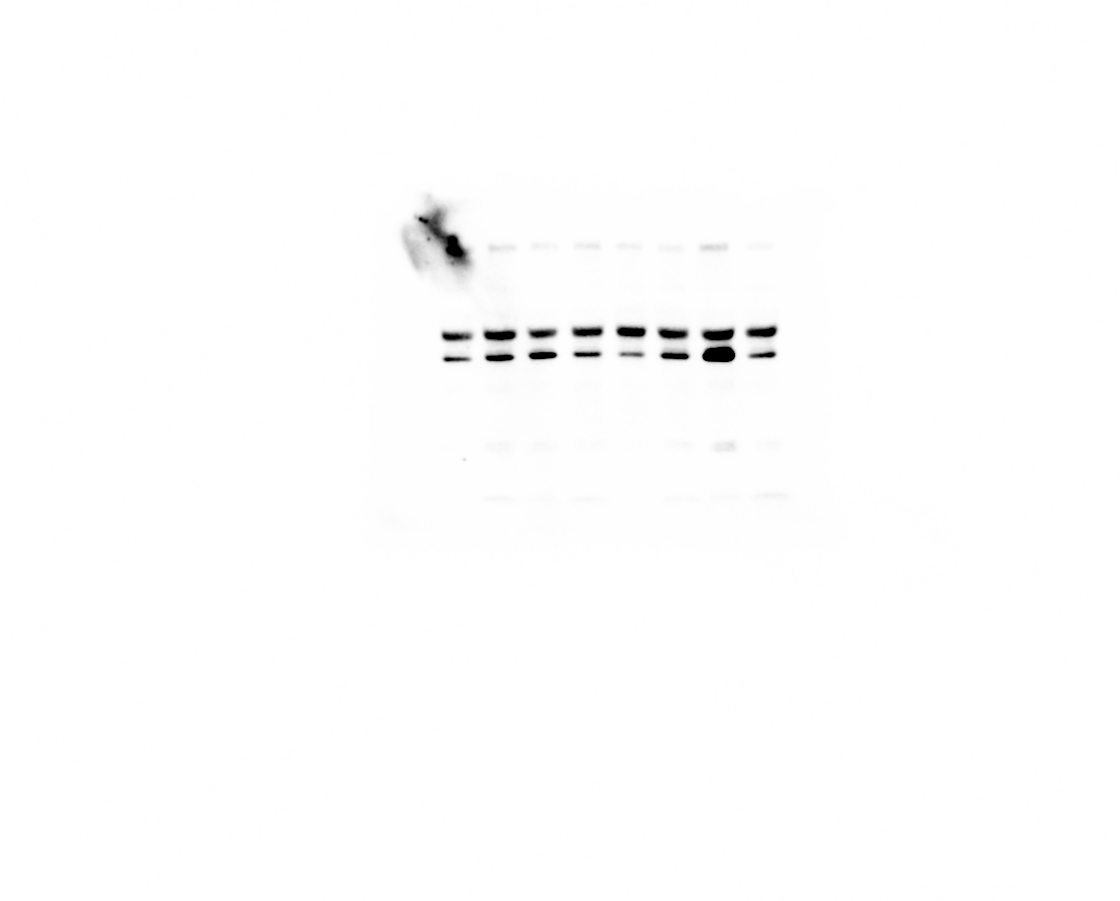

Supplement: Figure 3—source data 3. [file elife-89210-fig3-data3.zip › Figure 3- Source Data 3/individual pictures/ccasp8_5_min_exposure.jpg]

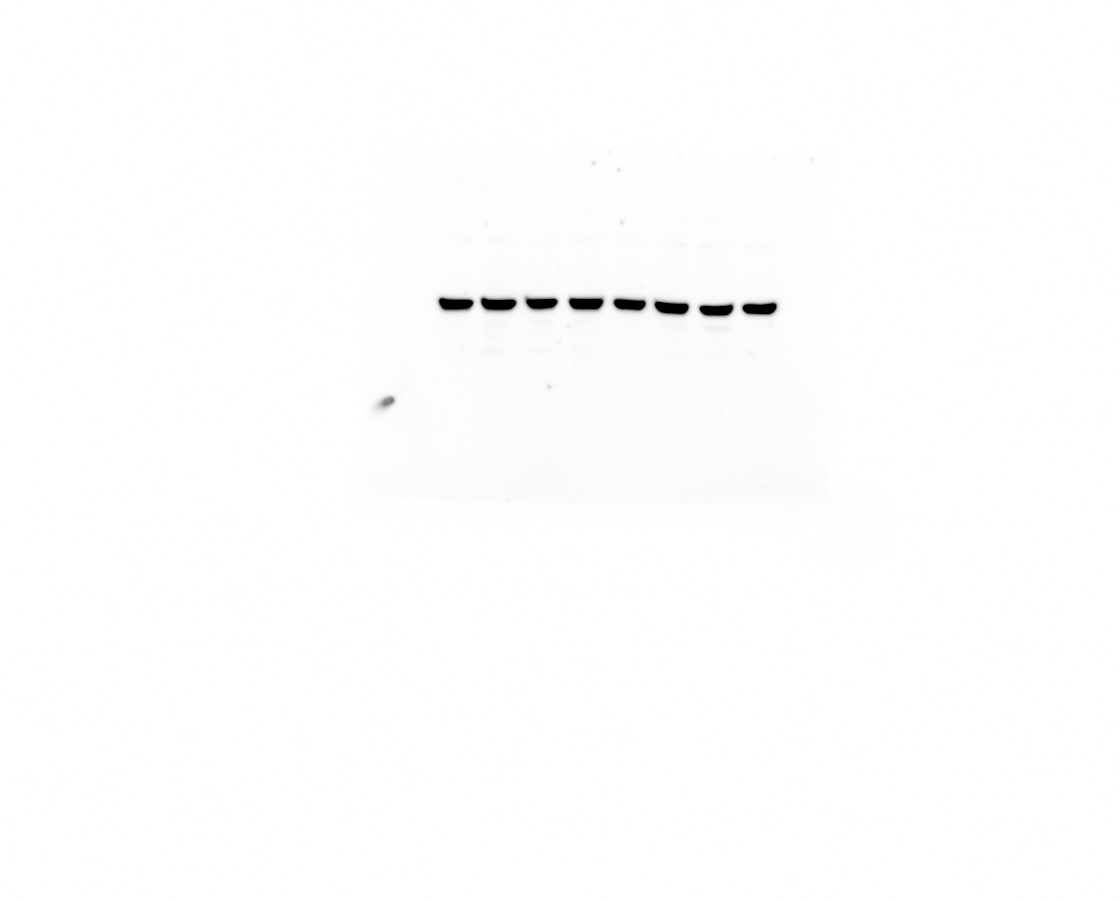

Supplement: Figure 3—source data 3. [file elife-89210-fig3-data3.zip › Figure 3- Source Data 3/individual pictures/GsdmD_5_min_exposure_gamma_onl.jpg]

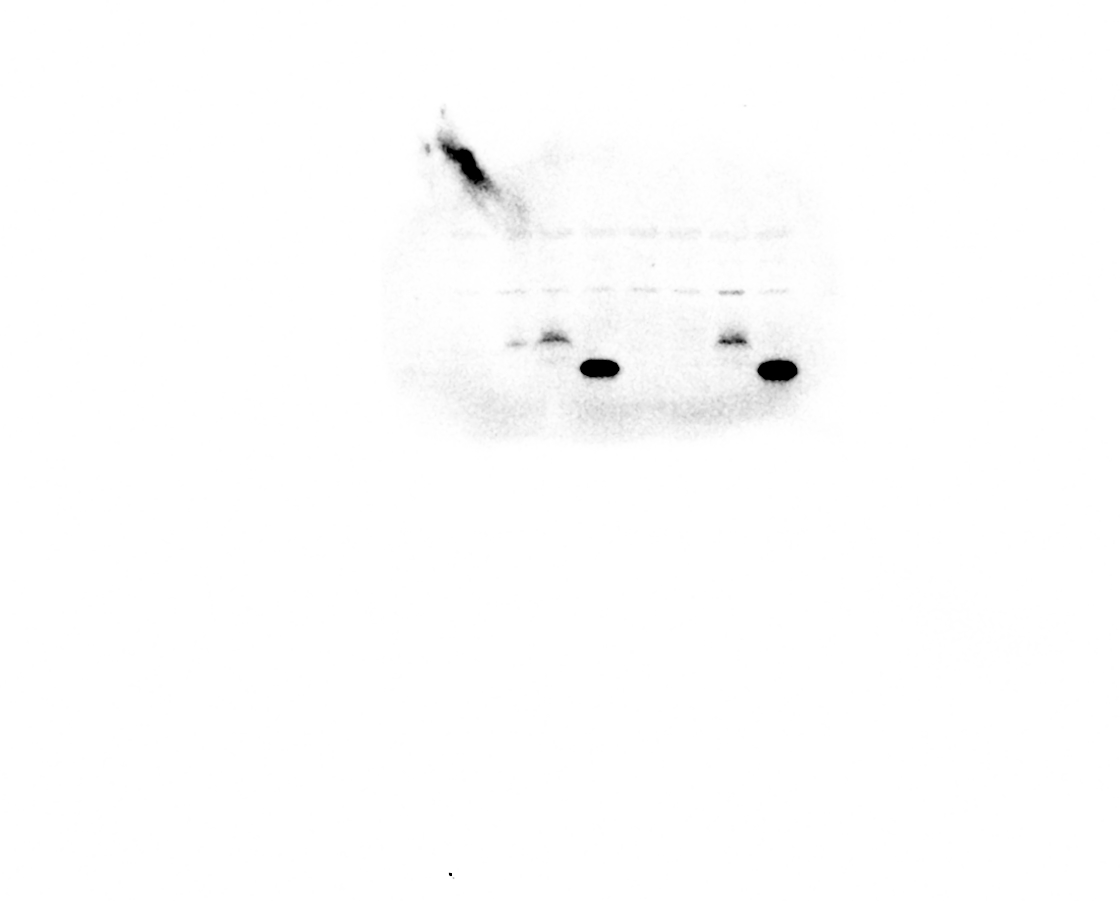

Supplement: Figure 3—source data 3. [file elife-89210-fig3-data3.zip › Figure 3- Source Data 3/individual pictures/HA_1_min_exposure_gamma_only.jpg]

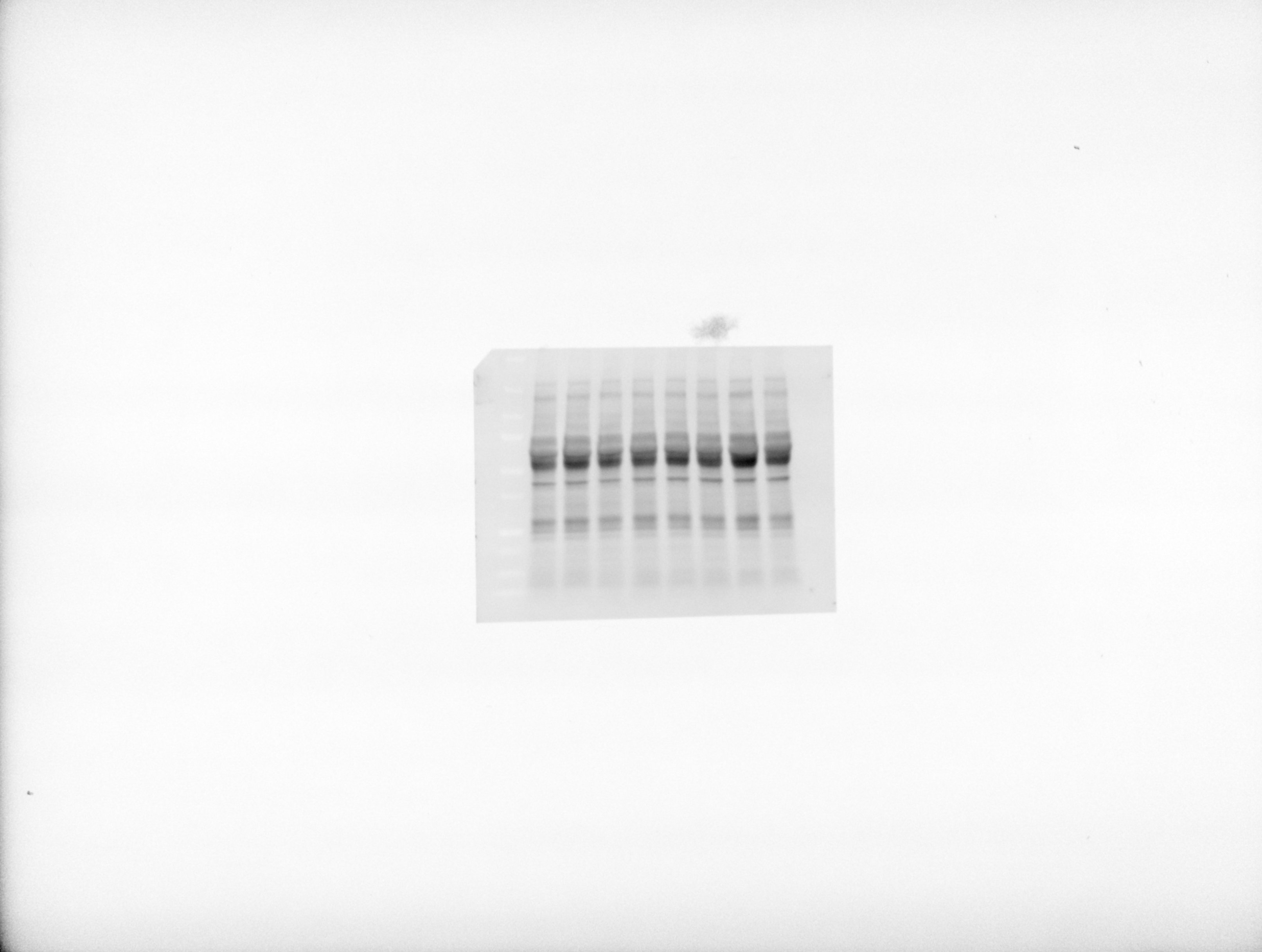

Supplement: Figure 3—source data 3. [file elife-89210-fig3-data3.zip › Figure 3- Source Data 3/individual pictures/membrane_total_protein.jpg]

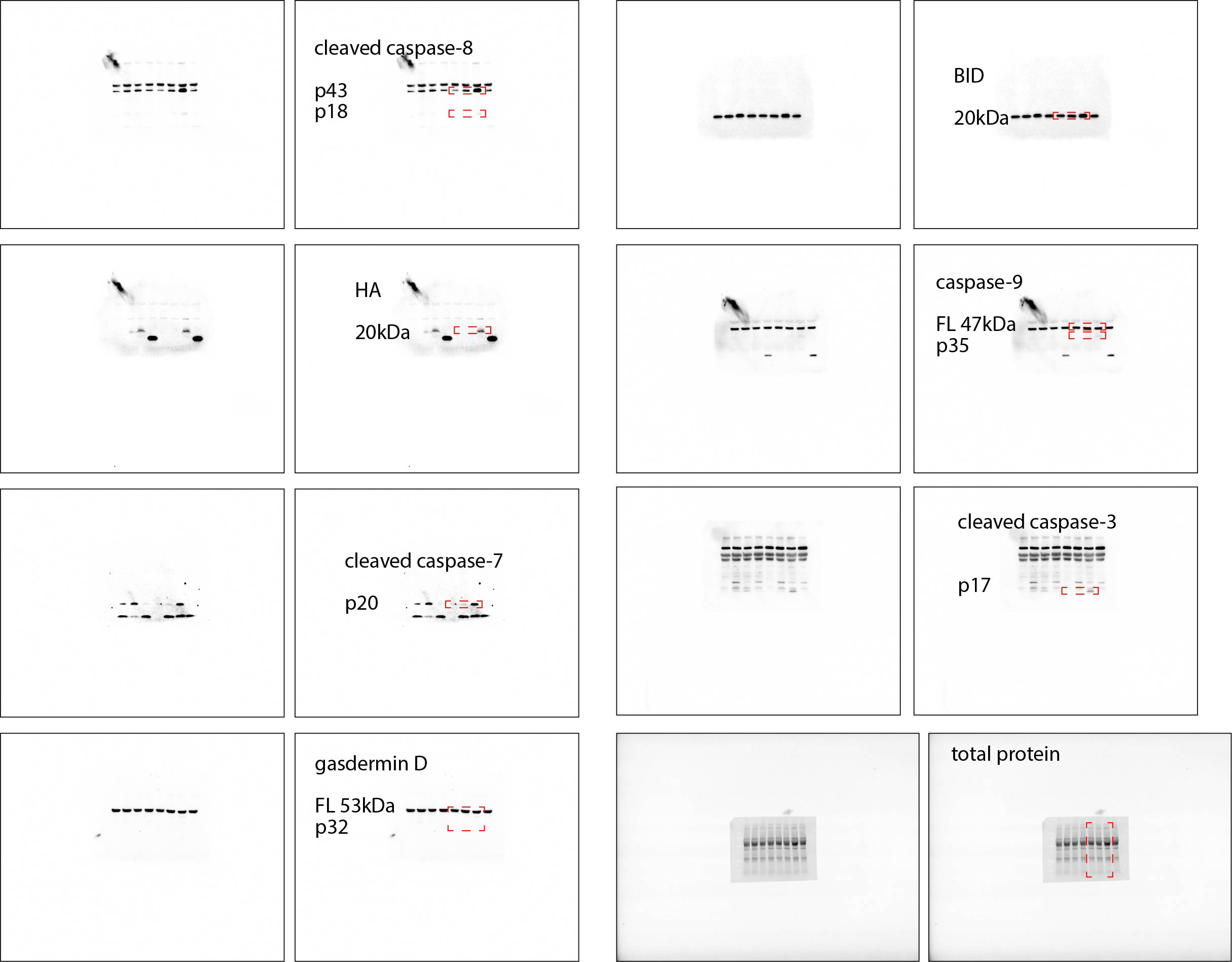

Supplement: Figure 3—source data 3. [file elife-89210-fig3-data3.zip › Figure 3- Source Data 3/western blots.jpg]

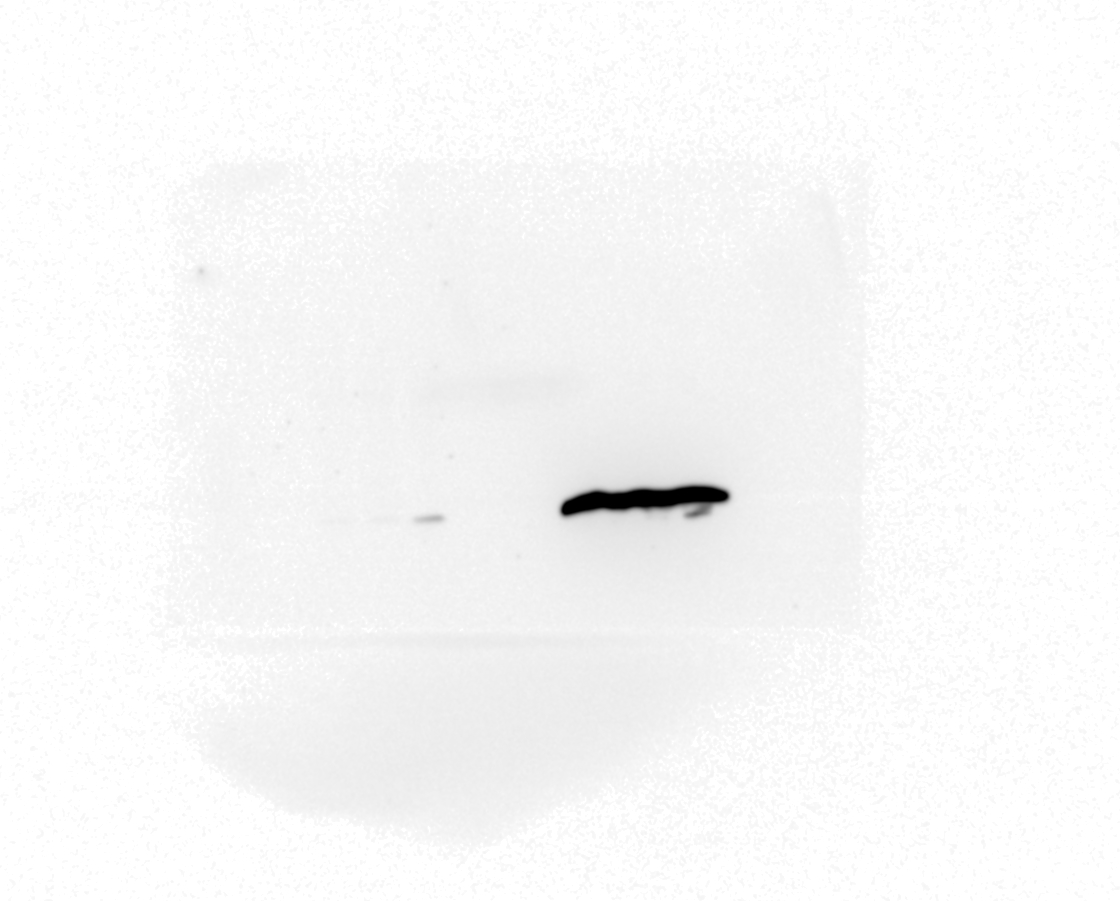

Supplement: Figure 3—source data 4. [file elife-89210-fig3-data4.zip › Figure 3- Source Data 4/individual pics/cyto_c_cyto_v_mito_10_min_.jpg]

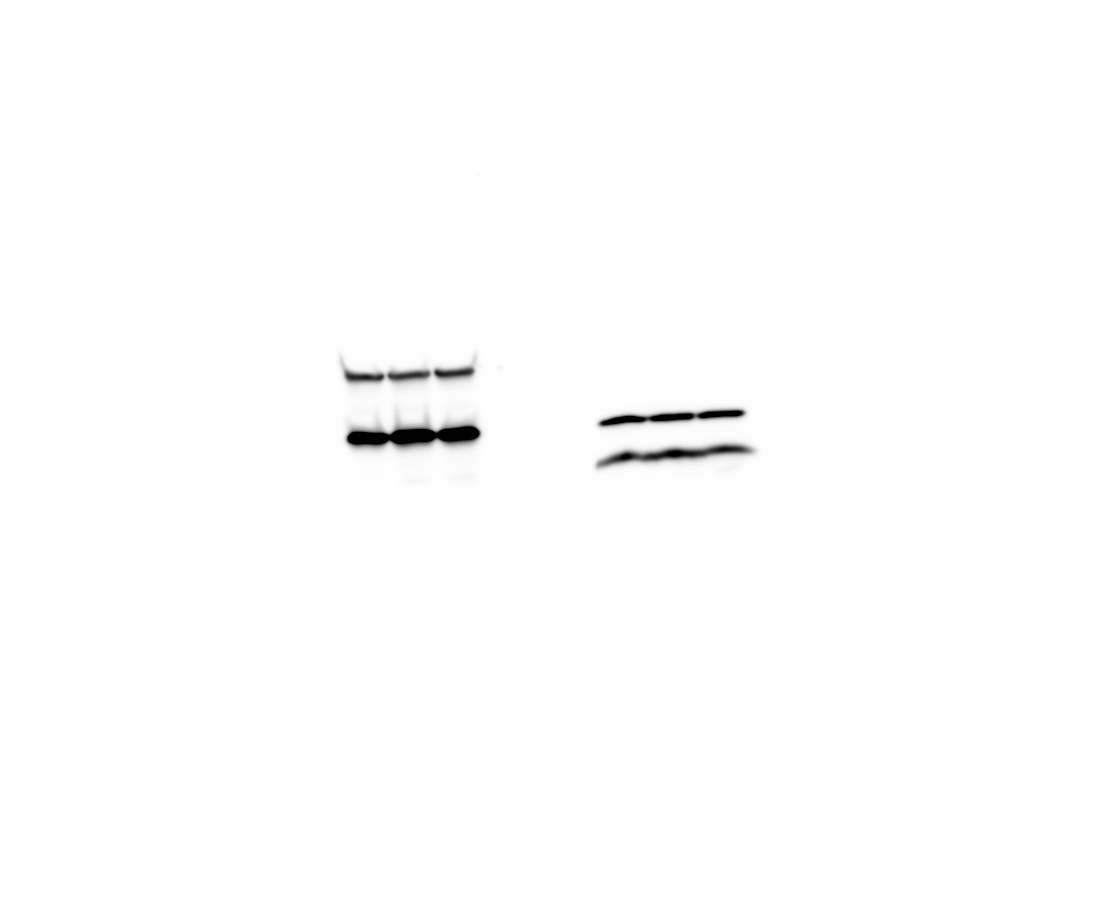

Supplement: Figure 3—source data 4. [file elife-89210-fig3-data4.zip › Figure 3- Source Data 4/individual pics/GAPDH_cyto_v_mito_1_min_ex.jpg]

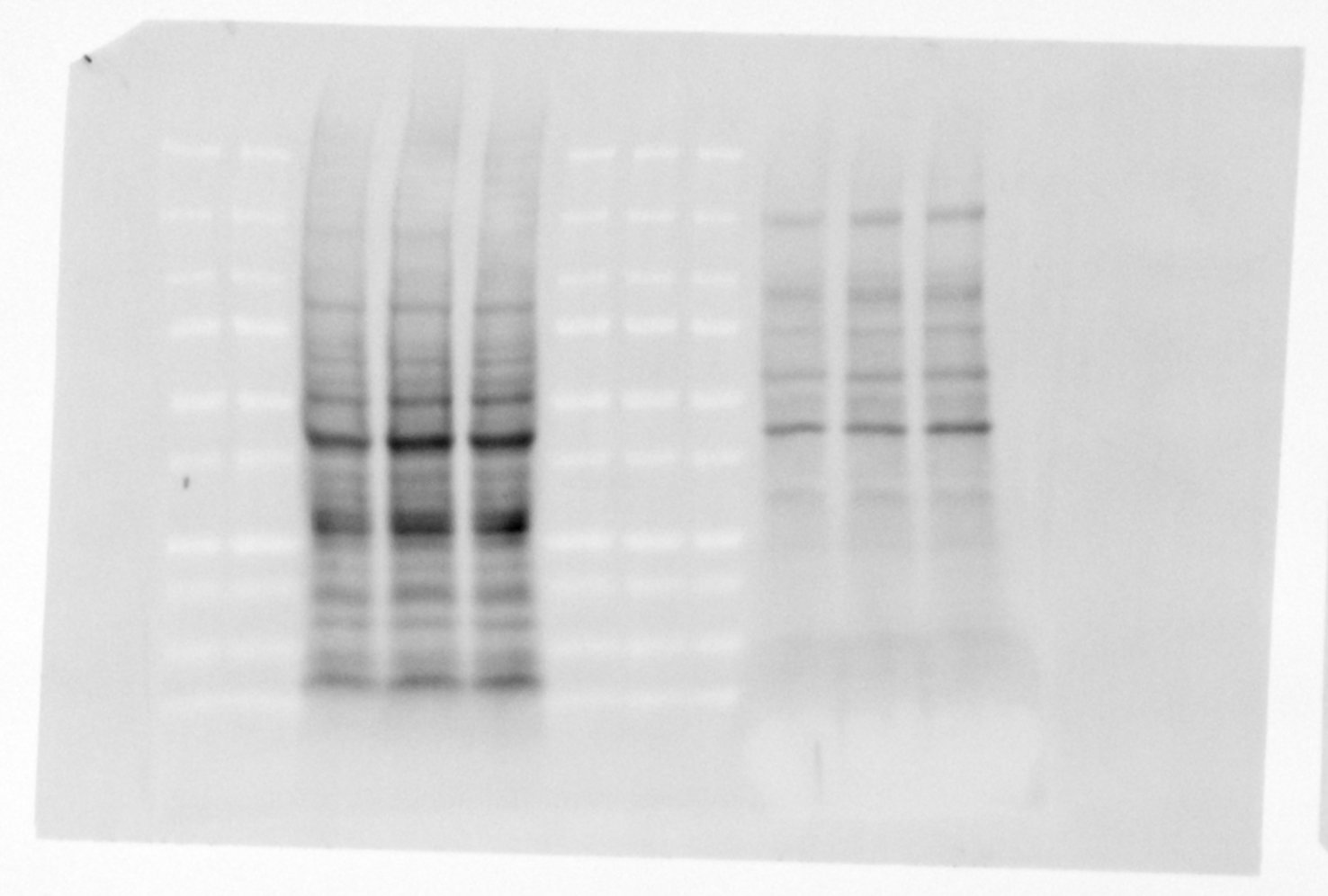

Supplement: Figure 3—source data 4. [file elife-89210-fig3-data4.zip › Figure 3- Source Data 4/individual pics/membrane_total_protein_cyto_v_.jpg]

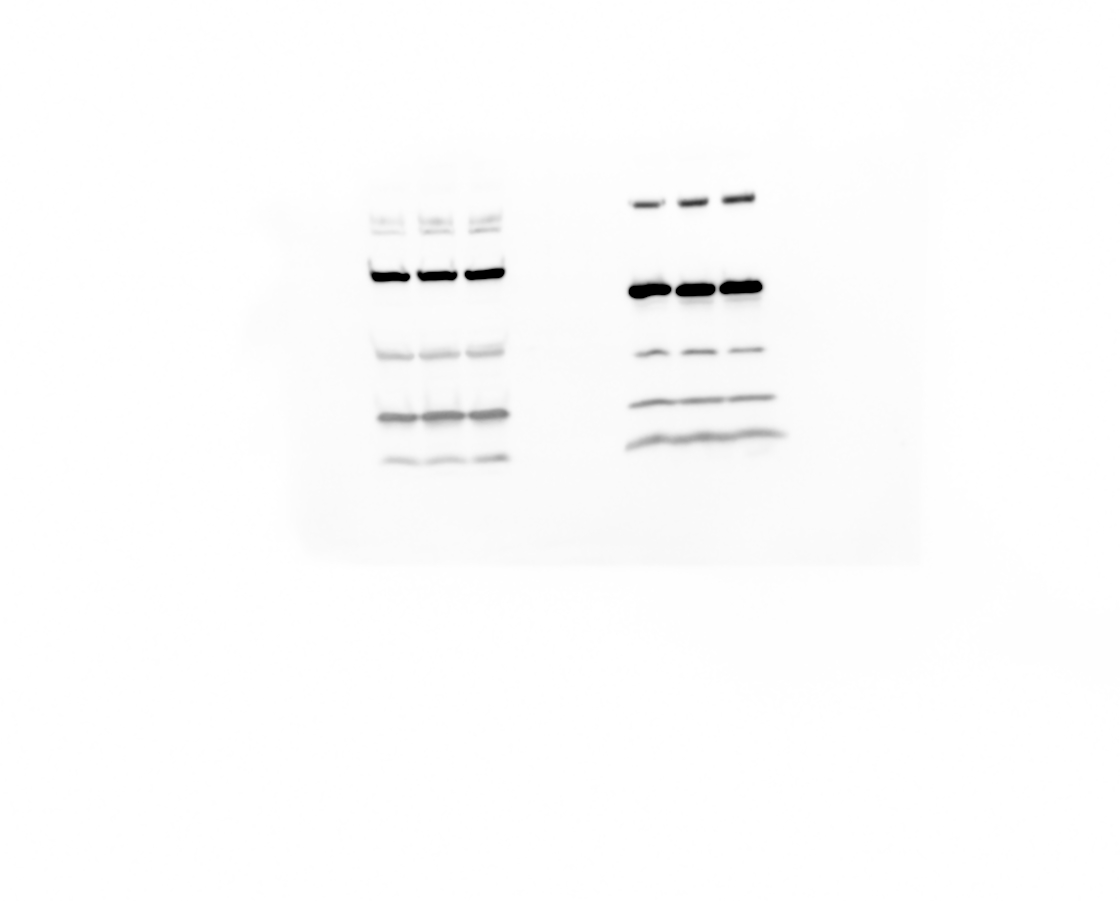

Supplement: Figure 3—source data 4. [file elife-89210-fig3-data4.zip › Figure 3- Source Data 4/individual pics/VDAC_cyto_v_mito_5_min_exp.jpg]

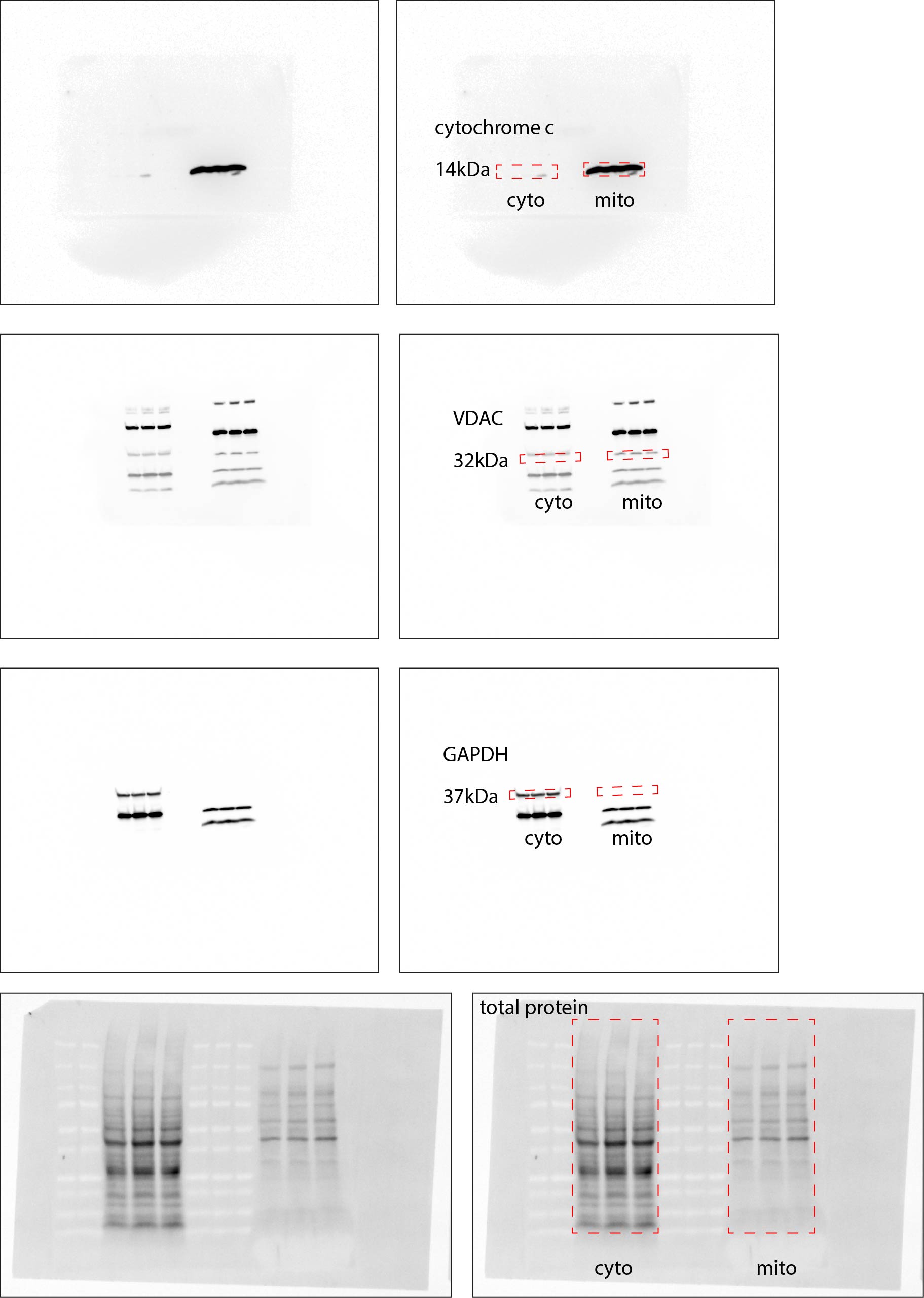

Supplement: Figure 3—source data 4. [file elife-89210-fig3-data4.zip › Figure 3- Source Data 4/western blots.jpg]

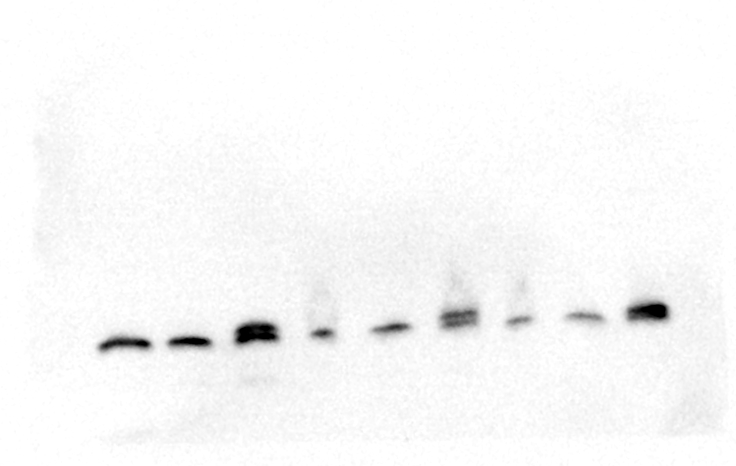

Supplement: Figure 3—figure supplement 1—source data 2. [file elife-89210-fig3-figsupp1-data2.zip › Figure 3- figure supplement 1- Source Data 1/individual pictures/BID_st.jpg]

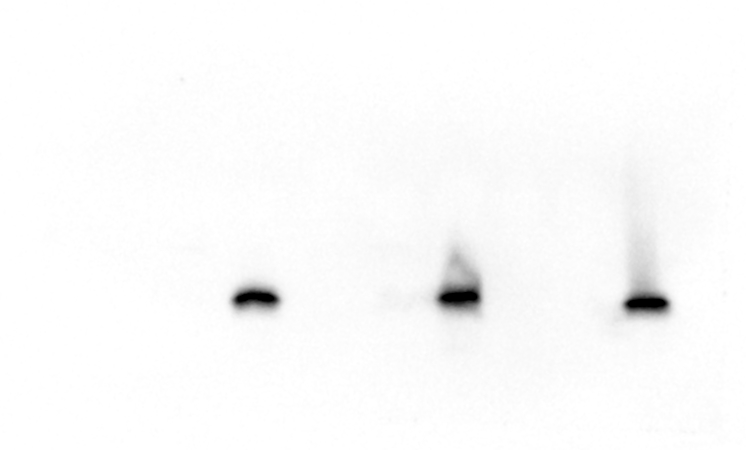

Supplement: Figure 3—figure supplement 1—source data 2. [file elife-89210-fig3-figsupp1-data2.zip › Figure 3- figure supplement 1- Source Data 1/individual pictures/HA_sta.jpg]

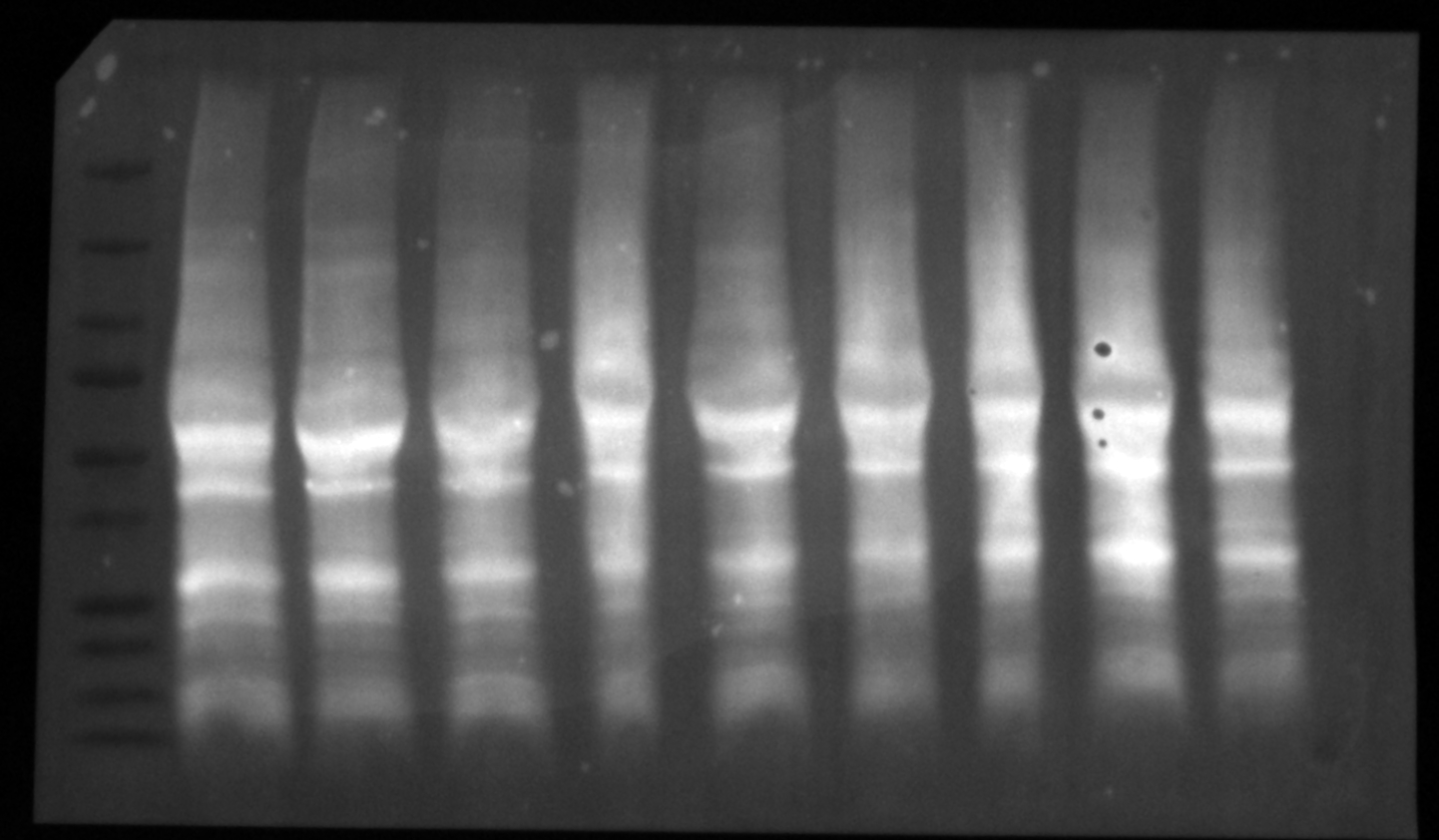

Supplement: Figure 3—figure supplement 1—source data 2. [file elife-89210-fig3-figsupp1-data2.zip › Figure 3- figure supplement 1- Source Data 1/individual pictures/protei.jpg]

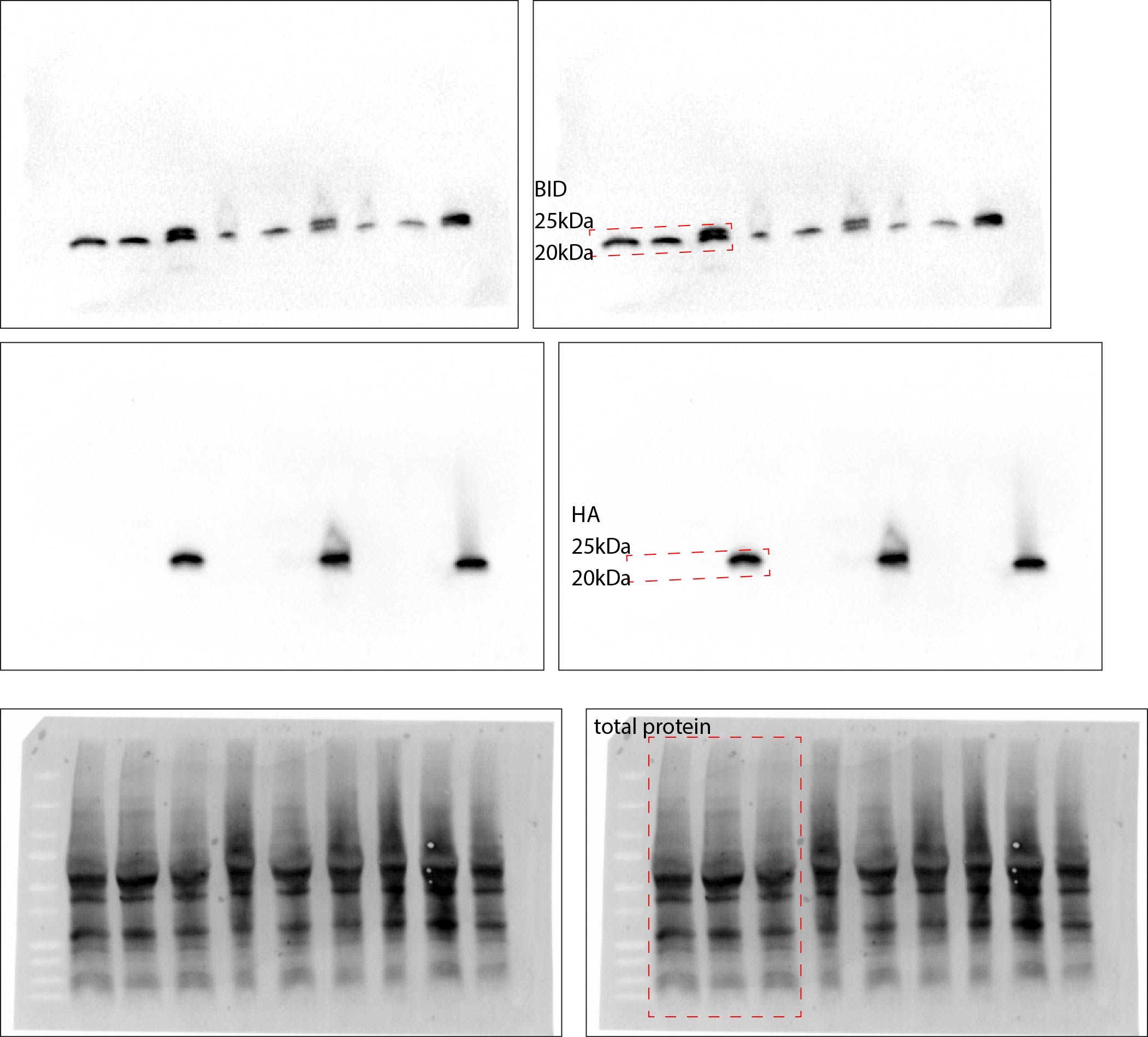

Supplement: Figure 3—figure supplement 1—source data 2. [file elife-89210-fig3-figsupp1-data2.zip › Figure 3- figure supplement 1- Source Data 1/western blots.jpg]

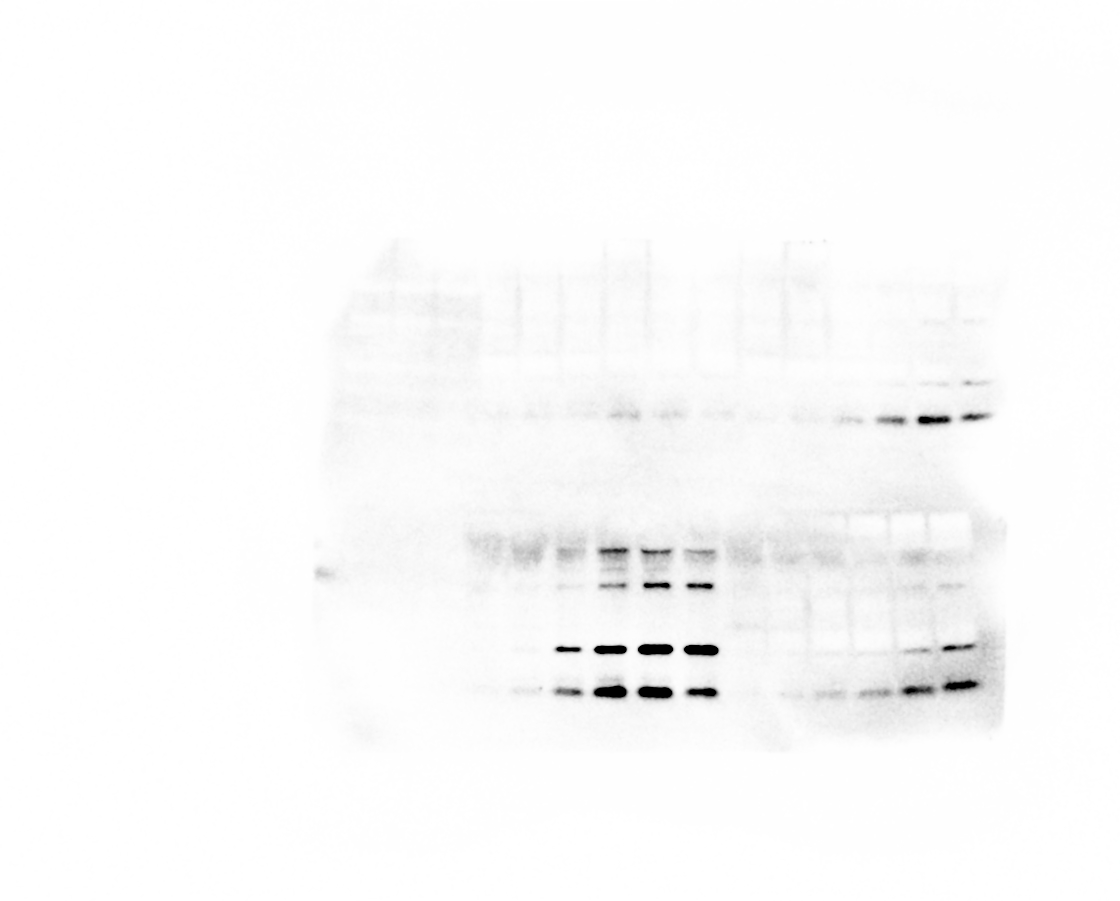

Supplement: Figure 4—source data 1. [file elife-89210-fig4-data1.zip › Figure 4- Source Data 1/individual pics/ccasp3_10_min_exposure_brighte.jpg]

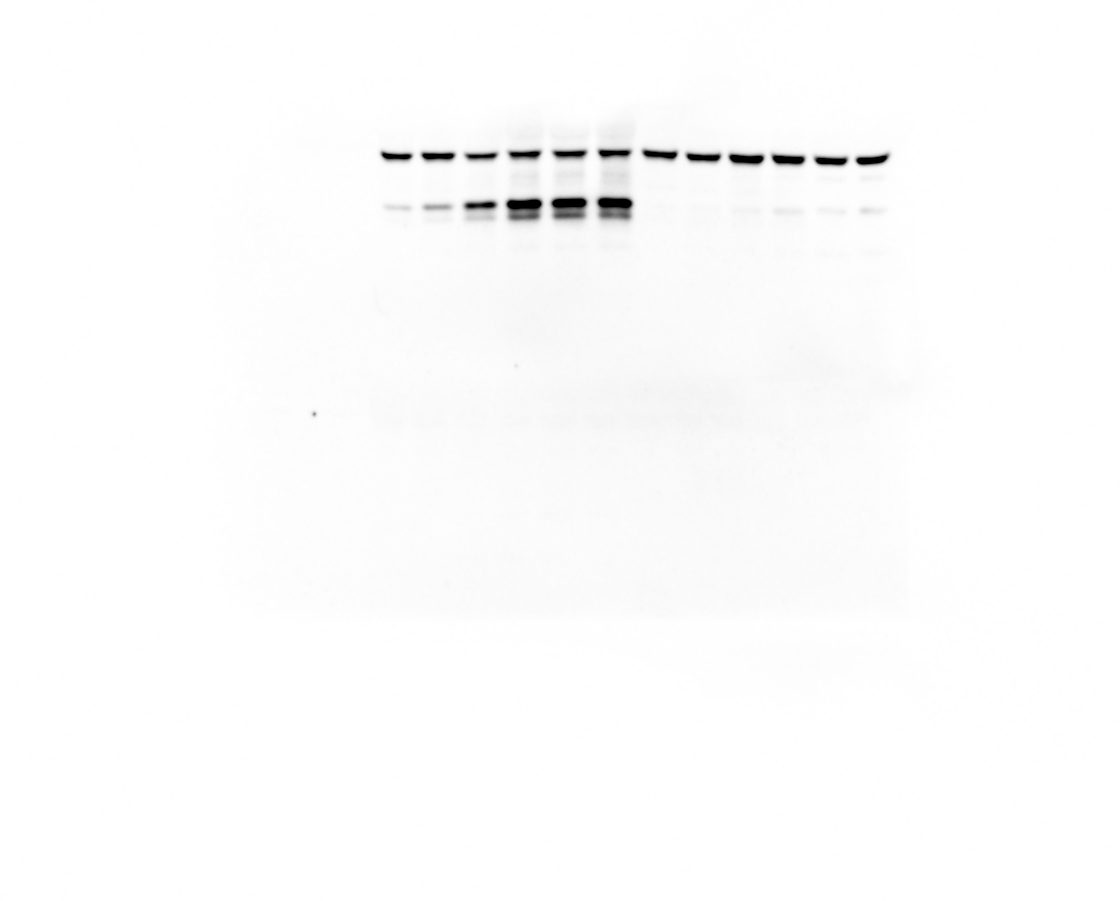

Supplement: Figure 4—source data 1. [file elife-89210-fig4-data1.zip › Figure 4- Source Data 1/individual pics/gsdmd_5_min_exposure_gamma_onl.jpg]

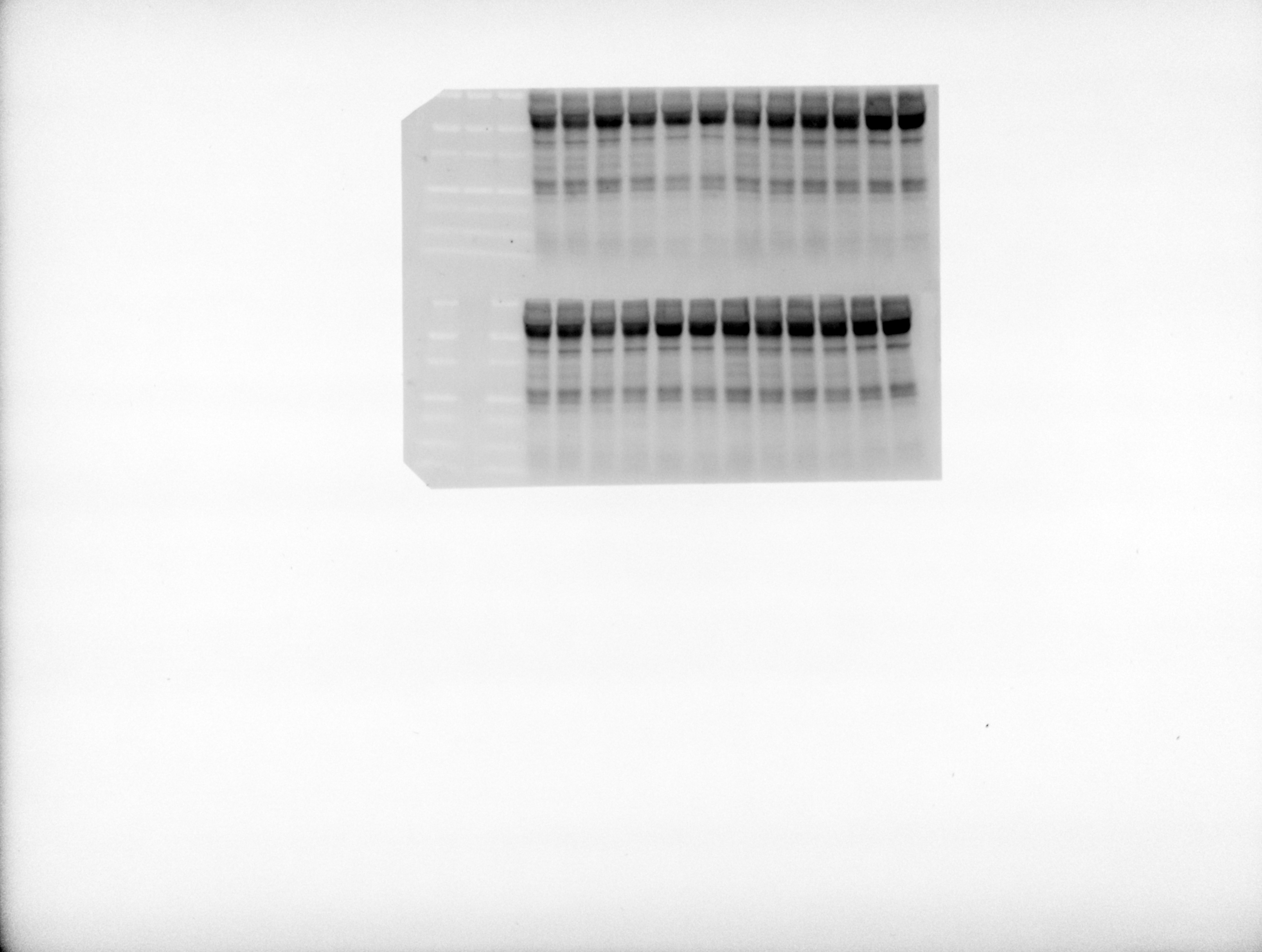

Supplement: Figure 4—source data 1. [file elife-89210-fig4-data1.zip › Figure 4- Source Data 1/individual pics/total_protein_membrane.jpg]

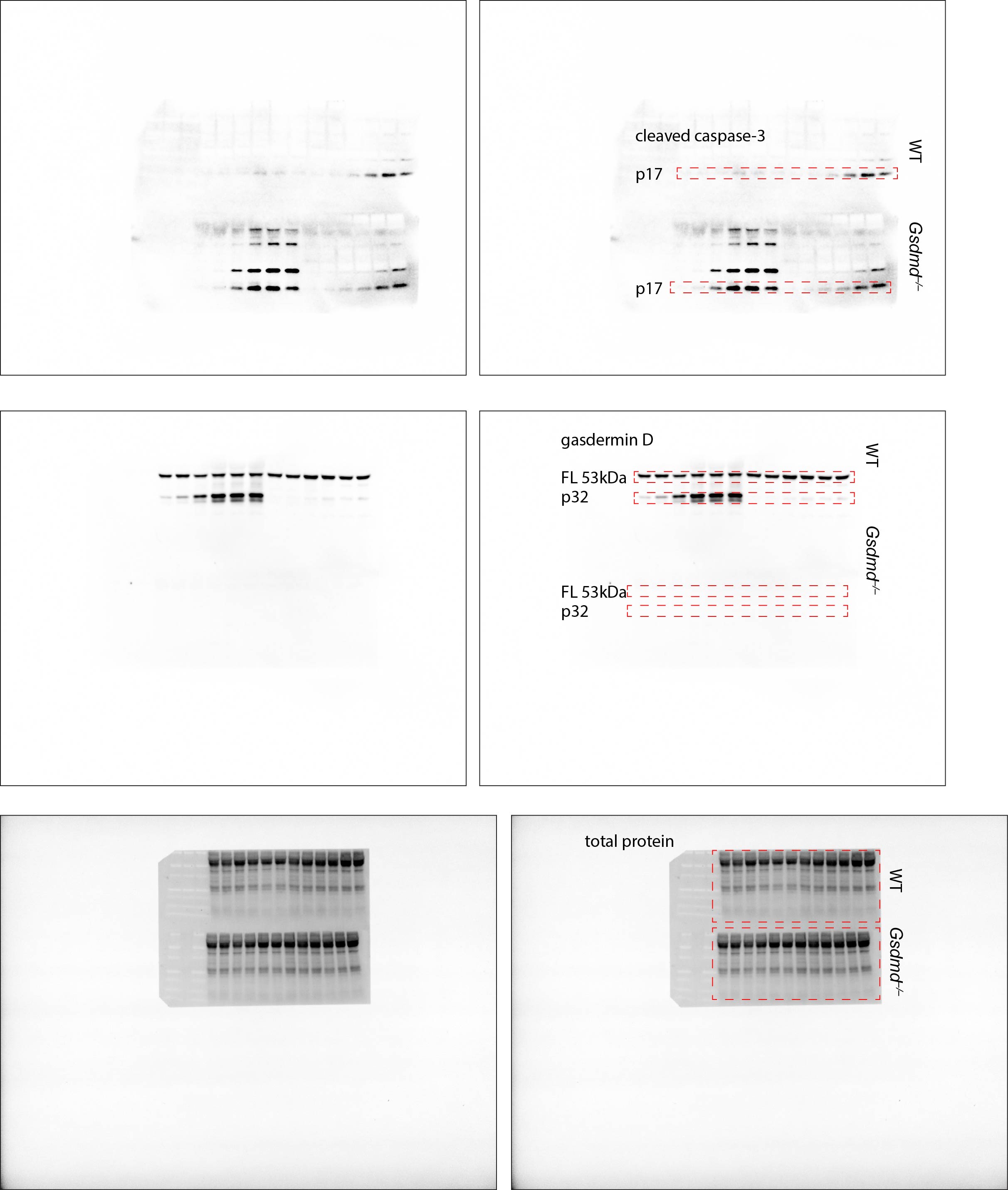

Supplement: Figure 4—source data 1. [file elife-89210-fig4-data1.zip › Figure 4- Source Data 1/western blots.jpg]
