# Supplementary material for: Apoptotic signaling clears engineered Salmonella in an organ-specific manner
Source: eLife. 2023 Dec 6;12:RP89210. doi: 10.7554/eLife.89210 (PMC10699806; doi:10.7554/eLife.89210)
Supplement: MDAR checklist [file elife-89210-mdarchecklist1.docx]

**Materials Design Analysis Reporting (MDAR)**

**Checklist for Authors**

The [MDAR framework](https://osf.io/xfpn4/) establishes a minimum set of requirements in transparent reporting mainly applicable to studies in the life sciences.

*eLife* asks authors to **provide detailed information within their article** to facilitate the interpretation and replication of their work. Authors can also upload supporting materials to comply with relevant reporting guidelines for health-related research (see [EQUATOR Network](http://www.equator-network.org/%20)), life science research (see the [BioSharing Information Resource](http://biosharing.org/)), or animal research (see the [ARRIVE Guidelines](http://www.plosbiology.org/article/info:doi/10.1371/journal.pbio.1000412) and the [STRANGE Framework](https://doi.org/10.1038/d41586-020-01751-5); for details, see *eLife*’s [Journal Policies](https://reviewer.elifesciences.org/author-guide/journal-policies)). Where applicable, authors should refer to any relevant reporting standards materials in this form.

For all that apply, please note **where in the article** the information is provided. Please note that we also collect information about data availability and ethics in the submission form.

**Materials:**

| **Newly created materials** | **Indicate where provided: section/figure legend** | **N/A** |
| --- | --- | --- |
| The manuscript includes a dedicated "materials availability statement" providing transparent disclosure about availability of newly created materials including details on how materials can be accessed and describing any restrictions on access. | Yes, under “Data, Materials, and Software Availability” subheading |  |
|  |  |  |
| **Antibodies** | **Indicate where provided: section/figure legend** | **N/A** |
| For commercial reagents, provide supplier name, catalogue number and [RRID](https://scicrunch.org/resources), if available. | Yes, in “Materials and Methods” and in “Key Resources Table” |  |
|  |  |  |
| **DNA and RNA sequences** | **Indicate where provided: section/figure legend** | **N/A** |
| Short novel DNA or RNA including primers, probes: Sequences should be included or deposited in a public repository. | Plasmid maps available by request, stored on Benchling. Noted in “Data, Materials, and Software Availability” |  |
|  |  |  |
| **Cell materials** | **Indicate where provided: section/figure legend** | **N/A** |
| Cell lines: Provide species information, strain. Provide accession number in repository OR supplier name, catalog number, clone number, OR RRID. |  | N/A |
| Primary cultures: Provide species, strain, sex of origin, genetic modification status. | Noted in “Materials and Methods” and in “Key Resources Table” |  |
|  |  |  |
| **Experimental animals** | **Indicate where provided: section/figure legend** | **N/A** |
| Laboratory animals or Model organisms: Provide species, strain, sex, age, genetic modification status. Provide accession number in repository OR supplier name, catalog number, clone number, OR RRID. | Noted in “Materials and Methods” and in “Key Resources Table” |  |
| Animal observed in or captured from the field: Provide species, sex, and age where possible. |  | N/A |
|  |  |  |
| **Plants and microbes** | **Indicate where provided: section/figure legend** | **N/A** |
| Plants: provide species and strain, ecotype and cultivar where relevant, unique accession number if available, and source (including location for collected wild specimens). |  | N/A |
| Microbes: provide species and strain, unique accession number if available, and source. | Noted in “Materials and Methods” and in “Key Resources Table” |  |
|  |  |  |
| **Human research participants** | **Indicate where provided: section/figure legend) or state if these demographics were not collected** | **N/A** |
| If collected and within the bounds of privacy constraints report on age, sex, gender and ethnicity for all study participants. |  | **N/A** |

**Design:**

| **Study protocol** | **Indicate where provided: section/figure legend** | **N/A** |
| --- | --- | --- |
| If the study protocol has been pre-registered, provide DOI. For clinical trials, provide the trial registration number OR cite DOI. |  | N/A |
|  |  |  |
| **Laboratory protocol** | **Indicate where provided: section/figure legend** | **N/A** |
| Provide DOI OR other citation details if detailed step-by-step protocols are available. |  | N/A |
|  |  |  |
| **Experimental study design (statistics details) *** | | |
| **For in vivo studies: State whether and how the following have been done** | **Indicate where provided: section/figure legend. If it could have been done, but was not, write “not done”** | **N/A** |
| Sample size determination:  Target sample size was 6 mice per group based on power analysis and historical trends in data variance, however smaller or larger group sizes were used sometimes due to mouse availability. 6 mice are the preferred number to be used per group based on power analysis using the two tailed Student's t-test. The following parameters based on our prior experiments were used in power analysis (we use the website "Handbook of Biological Statistics, by John H. McDonald for reference). In a prior experiment that is typical of infection models in our lab, we find that the standard deviation for bacterial counts in wr infections is 1404 CFU (data is presented as log transformation; log(1404) = 0.17). We assume the standard deviation for the test infection will be similar. We wish to detect differences with a p value of 0.05 (called alpha in the power analysis). The power of the experiment is the percent chance to find the indicated difference if it exists. We wish to have an 80 percent chance to find a 2 fold difference (log10(2) = 0.301) and a 99% chance to detect a 3-fold difference in the CFU (log10(3) = 0.477). We will have an equal number of mice in each group (sample size ratio Nl /N2). Key values in historical data that were used for this power analysis are as follows CFU/mg of spleen from a Salmonella infection: 4079, 6168, 3545, 5371, 1823, ,4963; average 4325; stdev 1404 (we use the log transformed data for the power analysis which are 3.61, 3.79, 3.55, 3.73, 3.26, 3.70; average 3.61; with a standard deviation of 0.17). The desired minimum difference for a 2 fold effect is 8650 (log transformed 3.94). However, when mouse numbers are limited, sometimes we use fewer mice, accepting that we may not detect more subtle differences between groups. When higher mouse numbers are available, we use larger numbers of mice to ensure detection of statistically significant differences. | Methods and Materials have the following text: “Details of sample size determination, randomization, and blinding can be found in the MDAR.” |  |
| Randomisation:  All samples were allocated into experimental groups in a non-biased manner. Covariant controls are not relevant because we use inbred mice in all experiments. For mice, sexes were evenly distributed between groups. | Methods and Materials have the following text: “Details of sample size determination, randomization, and blinding can be found in the MDAR.” |  |
| Blinding:  Experimental group assignments were not blinded because all mice are genetically identical (except for X and Y chromosomes, which were distributed equally between groups). | Methods and Materials have the following text: “Details of sample size determination, randomization, and blinding can be found in the MDAR.” |  |
| Inclusion/exclusion criteria:  If after harvest, zero CFUs were collected for all bacterial strains used in inoculum, that single mouse was considered “uninfected”, which could occur due to experimental error, and was excluded from the study. If even one CFU was present for any of the bacterial strains used in the inoculum, that mouse was considered “infected” and included in the study data. A total of four mice were excluded using this criteria: one *Nlrc4^–/–^* D1 mouse in Figure 2B & 2D, one D3 mouse from Figure 2- figure supplement 1A-B, and two mice from a single independent experiment in Figure 5A-B (one D2 BID^ON^ mouse and one D1 FliC^ON^ mouse). | Noted in “Mice and Mouse infections” |  |
|  |  |  |
| **Sample definition and in-laboratory replication** | **Indicate where provided: section/figure legend** | **N/A** |
| State number of times the experiment was replicated in the laboratory. | Included in figure legend |  |
| Define whether data describe technical or biological replicates. | Included in figure legend |  |
|  |  |  |
| **Ethics** | **Indicate where provided: section/submission form** | **N/A** |
| Studies involving human participants: State details of authority granting ethics approval (IRB or equivalent committee(s), provide reference number for approval. |  | N/A |
| Studies involving experimental animals: State details of authority granting ethics approval (IRB or equivalent committee(s), provide reference number for approval. | Included in Materials and Methods |  |
| Studies involving specimen and field samples: State if relevant permits obtained, provide details of authority approving study; if none were required, explain why. |  | N/A |
|  |  |  |
| **Dual Use Research of Concern (DURC)** | **Indicate where provided: section/submission form** | **N/A** |
| If study is subject to dual use research of concern regulations, state the authority granting approval and reference number for the regulatory approval. |  | N/A |

**Analysis:**

| **Attrition** | **Indicate where provided: section/figure legend** | **N/A** |
| --- | --- | --- |
| Describe whether exclusion criteria were pre-established. Report if sample or data points were omitted from analysis. If yes, report if this was due to attrition or intentional exclusion and provide justification. | Included in methods and materials |  |
|  |  |  |
| **Statistics** | **Indicate where provided: section/figure legend** | **N/A** |
| Describe statistical tests used and justify choice of tests. | Included in figure legends and in Materials and Methods |  |
|  |  |  |
| **Data availability** | **Indicate where provided: section/submission form** | **N/A** |
| For newly created and reused datasets, the manuscript includes a data availability statement that provides details for access (or notes restrictions on access). |  | N/A |
| When newly created datasets are publicly available, provide accession number in repository OR DOI and licensing details where available. |  | N/A |
| If reused data is publicly available provide accession number in repository OR DOI, OR URL, OR citation. |  | N/A |
|  |  |  |
| **Code availability** | **Indicate where provided: section/figure legend** | **N/A** |
| For any computer code/software/mathematical algorithms essential for replicating the main findings of the study, whether newly generated or re-used, the manuscript includes a data availability statement that provides details for access or notes restrictions. |  | N/A |
| Where newly generated code is publicly available, provide accession number in repository, OR DOI OR URL and licensing details where available. State any restrictions on code availability or accessibility. |  | N/A |
| If reused code is publicly available provide accession number in repository OR DOI OR URL, OR citation. |  | N/A |

**Reporting:**

The MDAR framework recommends adoption of discipline-specific guidelines, established and endorsed through community initiatives.

| **Adherence to community standards** | **Indicate where provided: section/figure legend** | **N/A** |
| --- | --- | --- |
| State if relevant guidelines (e.g., ICMJE, MIBBI, ARRIVE, STRANGE) have been followed, and whether a checklist (e.g., CONSORT, PRISMA, ARRIVE) is provided with the manuscript. |  | N/A |

* We provide the following guidance regarding transparent reporting and statistics; we also refer authors to [Ten common statistical mistakes to watch out for when writing or reviewing a manuscript](https://doi.org/10.7554/eLife.48175).

**Sample-size estimation**

- You should state whether an appropriate sample size was computed when the study was being designed
- You should state the statistical method of sample size computation and any required assumptions
- If no explicit power analysis was used, you should describe how you decided what sample (replicate) size (number) to use

**Replicates**

- You should report how often each experiment was performed
- You should include a definition of biological versus technical replication
- The data obtained should be provided and sufficient information should be provided to indicate the number of independent biological and/or technical replicates
- If you encountered any outliers, you should describe how these were handled
- Criteria for exclusion/inclusion of data should be clearly stated
- High-throughput sequence data should be uploaded before submission, with a private link for reviewers provided (these are available from both GEO and ArrayExpress)

**Statistical reporting**

- Statistical analysis methods should be described and justified
- Raw data should be presented in figures whenever informative to do so (typically when N per group is less than 10)
- For each experiment, you should identify the statistical tests used, exact values of N, definitions of center, methods of multiple test correction, and dispersion and precision measures (e.g., mean, median, SD, SEM, confidence intervals; and, for the major substantive results, a measure of effect size (e.g., Pearson's r, Cohen's d)
- Report exact p-values wherever possible alongside the summary statistics and 95% confidence intervals. These should be reported for all key questions and not only when the p-value is less than 0.05.

**Group allocation**

- Indicate how samples were allocated into experimental groups (in the case of clinical studies, please specify allocation to treatment method); if randomization was used, please also state if restricted randomization was applied
- Indicate if masking was used during group allocation, data collection and/or data analysis
